# Supplementary material for: The atlas of unburnable oil for supply-side climate policies
Source: Nat Commun. 2024 Mar 14;15:2318. doi: 10.1038/s41467-024-46340-6 (PMC10940309; doi:10.1038/s41467-024-46340-6)
Supplement: Supplementary file 1 — Suplementary Information [file 41467_2024_46340_MOESM1_ESM.pdf]

Supplementary Information

The atlas of unburnable oil for supply-side climate policies

The atlas of unburnable oil for supply-side climate policies

Lorenzo Pellegrini[1†], Murat Arsel[1], Gorka Muñoa[2, 3], Guillem Rius-Taberner[2, 3], Carlos Mena[4], Martí Orta-Martínez[2, 3 * †]

[1] International Institute of Social Studies (ISS), Erasmus University Rotterdam.

[2] Departament de Biologia Evolutiva, Ecologia i Ciències Ambientals, Facultat de Biologia, Universitat de Barcelona, 08028 Barcelona, Catalonia.

[3] Institute de Recerca de la Biodiversitat (IRBio), Universitat de Barcelona, Catalonia.

[4] Institute of Geography, Universidad San Francisco de Quito, Quito, Ecuador.

[*] Corresponding author: Marti.Orta@ub.edu

[†] These authors contributed equally to this work

Supplementary Information

Buffer distance based on health risks associated with oil extraction

Setting a safe distance from oil extraction with respect to health risks is not an easy task. Health effects among people residentially exposed to upstream oil industry contaminants have been poorly studied, particularly in low- and middle-income countries (LMICs)[1]. In a systematic review, O'Callaghan-Gordo et al.[1] only found 11 studies examining potential health effects of exposed communities in LMICs. 10 of these studies were conducted in the Ecuadorian and Peruvian Amazon and one in the Niger Delta. The development of unconventional oil and gas projects and the dramatic increase of fracking in the USA in the past decade started to create concern among environmental health researchers[2] and more environmental health studies are now available regarding fracking in high income countries[3,4]. Besides the lack of scientific data, the use of sub-standard technologies for oil extraction in LMICs adds a further twist in the difficult task of setting a unique safe distance from oil extraction[5.] Similarly, the different environmental characteristics of the oil extraction locations around the globe makes the identification of a uniform safe distance difficult. Globally, there are an estimated 70,000 oil fields across ~100 countries[3] .In the case of shale gas drilling, Texas (USA) permits drilling 200 ft (approx. 60 m) from residences. However, many municipalities in Texas have established longer setback distances (setback distances from residences range from 300 to 1500 ft, approx. 90 to 460 m)[6]. Setback distances in Texas have increased over time, and, according to Fry, rather than technically-based, setbacks are political compromises: "rigorous and empirical research was not utilized to determine or demarcate 'safe' or 'healthy' distances, and the setback distances are highly politicized compromises between residents' concerns about the proximity of gas wells to their homes, mineral owners' rights to profit from gas drilling, and the city council's fear of legal lawsuits for a regulatory takings"[6]. In fact, McKenzie et al.[7] found that residents living within 0.8 km from a fracking well are at a higher health risk than those farther away with benzene as the major contributor to the risk. Coons e al.[8] also found that significant ambient benzene emissions exist within close proximity to a fracking well (0.8 km), which resulted in significant public health problems. Methane concentrations in drinking water wells within 1 km of a fracking well can reach potentially explosive levels[9]. A recent working paper suggested that a distance within at least 2.5 km from a gas well is detrimental to fetus development due to exposure to shale gas extraction[10]. In the USA, a proximity within 1 km to a fracking well is a recurring critical value used for significant risks to the environment and public health. Ref[11]used the distances 1 km, 2 km, and 3 km as break values to group risk levels into high (≤1 km), moderate (1–2 km), and low (2–3 km) risks. Beyond 3 km, Meng[11] assumed there was little impact of fracking wells on the environment and inhabitants in the USA. However, a much more recent review of epidemiological research on

unconventional oil and gas development and health outcomes (all of them conducted in the USA or Australia) showed detectable health effects in distances of up to 10 miles (aprox. 16km) [4].

In the very few studies that exist in LMICs, larger distances are suggested. O'Callaghan-Gordo et al.[12], found that increased distance between residence and an oil processing facility (i.e. oil wells, central production facilities, gathering stations and pumping stations) in the Peruvian Amazon was associated with lower Blood Lead Levels (BLL)[12]. The median distance from the studied communities to a processing facility was 5.5km, and BLL decreased 5% every 10km up to 200km distance from the oil facilities. A study in south-eastern Bolivia that measured the levels of total petroleum hydrocarbons (TPH), polycyclic aromatic hydrocarbons (PAH), and 22 metals in the drinking water of residents living ≤30 km from an oil extraction field found that three-quarters of the samples were contaminated with concentrations exceeding the reference levels[13]. Similarly, a meta-analysis of chemical data from governmental institutions and oil company reports proved that the dumping of produced water (i.e. the main waste product of oil extraction operations) increased lead, cadmium, chromium and barium concentrations in rivers of the Peruvian Amazon up to 36km downstream of the oil facilities[14].

Differences of unburnable regional oil estimates between studies

When compared to the regional distribution of unburnable conventional resources proposed by Welsby et al.[15] , the exclusion zones of this study produce lower proportions of unburnable conventional oil resources (as expected since globally they also concern less resources) with the greatest difference for Canada (2% and 82% unburnable conventional resources in our analysis and Welsby et al. 2021, respectively) and the exception of Developing Asia (outside of China and India; 78% and 48% unburnable conventional resources in our analysis and Welsby et al. 2021, respectively). The regions with large differences are indicative of a divergence between the socio-environmental criteria we employed to identify unburnable resources and the economic ones used by Welsby et al. 2021.

Supplementary Table 1. Burnable and unburnable fossil fuels by 2050 based on data from Welsby et al.[15] .This distribution of unburnable fossil fuels categories is based on the supply costs of different fossil fuel resources, taking into account extraction, refining, and transport costs, and using a remaining carbon budget of 580 GtCO2 under a global 1.5°C target[15]. Oil volumes are provided in giga-barrels (Gbbl), gas in cubic terameters (Tm^3) and coal in gigatons (Gt).

			Burnable				Unburnable			
						GtCO2				GtCO2
Reserves	Oil	Total	539	Gbbl	42%	232	744	Gbbl	58%	320
	Gas		64	Tm^3	41%	124	92	Tm^3	59%	178
	Coal		102	Gt	11%	204	826	Gt	89%	1653
Resources	Oil	Conventional	752	Gbbl	29%	323	1823	Gbbl	71%	784
		Unconventional	11	Gbbl	1%	5	1513	Gbbl	99%	651
		Total	783	Gbbl	19%	336	3336	Gbbl	81%	1434
	Gas	Conventional	79	Tm^3	19%	152	341	Tm^3	81%	661
		Unconventional	22	Tm^3	7%	42	284	Tm^3	93%	551
		Total	102	Tm^3	14%	197	625	Tm^3	86%	1212
	Coal		122	Gt	3%	245	3960	Gt	97%	7927

Supplementary Table 2. List of countries in each of the 10 regions presented. Extracted from Welsby et al.[15].

**Region**	**Countries**
**Africa**	Algeria, Angola, Benin, Botswana, Burkina Faso, Burundi, Cameroon, Cape Verde, Central African Republic, Chad, Comoros, Congo, Côte d'Ivoire, Democratic Republic of the Congo, Djibouti, Egypt, Equatorial Guinea, Eritrea, Ethiopia, Gabon, Gambia, Ghana, Guinea, Guinea-Bissau, Kenya, Lesotho, Liberia, Libya, Madagascar, Malawi, Mali, Mauritania, Morocco, Mozambique, Namibia, Niger, Nigeria, Rwanda, Sao Tome and Principe, Senegal, Seychelles, Sierra Leone, Somalia, South Africa, South Sudan, Sudan, Swaziland, Togo, Tunisia, Uganda, United Republic of Tanzania, Zambia, Zimbabwe
**Australia and other OECD Pacific**	Australia, Japan, New Zealand, Republic of Korea
**Canada**	Canada
**China and India**	China, Taiwan, Tibet, India
**Russia and former Soviet states**	Armenia, Azerbaijan, Belarus, Estonia, Georgia, Kazakhstan, Kyrgyzstan, Latvia, Lithuania, Republic of Moldova, Russian Federation, Tajikistan, Turkmenistan, Ukraine, Uzbekistan
**Central and South America**	Anguilla, Antigua and Barbuda, Argentina, Aruba, Bahamas, Barbados, Belize, Bermuda, Bolivia, Brazil, Cayman Islands, Chile, Colombia, Costa Rica, Cuba, Dominica, Dominican Republic, Ecuador, El Salvador, Falkland Islands, Grenada, Guatemala, Guyana, Haiti, Honduras, Jamaica, Martinique, Mexico, Netherlands Antilles, Nicaragua, Panama, Paraguay, Peru, Saint Kitts and Nevis, Saint Lucia, Saint Vincent and the Grenadines, Suriname, Trinidad and Tobago, Uruguay, Venezuela (Bolivarian Republic of)
**Europe**	Albania, Andorra, Austria, Belgium, Denmark, Faroe Islands, Finland, France, Germany, Gibraltar, Greece, Greenland, Iceland, Ireland, Italy, Luxembourg, Malta, Monaco, Netherlands, Norway, Portugal, San Marino, Spain, Sweden, Switzerland, United Kingdom, Vatican, Bosnia and Herzegovina, Bulgaria, Croatia, Czech Republic, Hungary, Montenegro, Poland, Romania, Serbia, Slovakia, Slovenia, The former Yugoslav Republic of Macedonia
**Middle East**	Bahrain, Brunei Darussalam, Cyprus, Iran (Islamic Republic of), Israel, Jordan, Kuwait,

Region	Countries
	Lebanon, Occupied Palestinian Territory, Oman, Qatar, Saudi Arabia, Syrian Arab Republic, Turkey, United Arab Emirates, Yemen
**Other Developing Asia**	Afghanistan, American Samoa, Bangladesh, Bhutan, Cambodia, Democratic People's Republic of Korea, Fiji, French Polynesia, Indonesia, Kiribati, Laos, People's Democratic Republic, Malaysia, Maldives, Mauritius, Mongolia, Myanmar, Nepal, New Caledonia, Pakistan, Papua New Guinea, Philippines, Samoa, Singapore, Solomon Islands, Sri Lanka, Thailand, Timor-Leste, Tonga, Vanuatu, Vietnam
**USA**	United States of America

Supplementary Table 3. List of top sedimentary basins containing the highest amount of unburnable conventional oil resources according to socio-environmental criteria. Cumulative percentage of unburnable resources and intersected area per each sedimentary basin is provided. Volumes are given in giga-barrels (thousand million oil barrels, Gbbl).

Sedimentary Basins	Oil resources overlapped (Gbbl)	Cumulative top-priority unburnable oil resources (%)	Intersected area (km2)	Cumulative area intersected (km2)	Cumulative area intersected (%)
Zagros Fold Belt	70.79	12%	234,465	234,465	1%
Gulf Coast Basins	59.73	21%	598,505	832,970	3%
Mesopotamian Foredeep Basin	58.07	31%	61,136	894,107	3%
Maracaibo Basin	21.93	35%	44,009	938,116	3%
West Siberian Basin	21.84	38%	259,254	1,197,369	4%
Rub Al Khali Basin	16.78	41%	108,623	1,305,993	4%
Northern Alaska	14.99	43%	137,977	1,443,970	5%
Greater Ghawar Uplift	14.41	46%	12,864	1,456,834	5%
Villahermosa Uplift	13.48	48%	12,686	1,469,519	5%
Niger Delta	12.88	50%	90,990	1,560,509	5%

Sedimentary Basins	Oil resources overlapped (Gbbl)	Cumulative top-priority unburnable oil resources (%)	Intersected area (km2)	Cumulative area intersected (km2)	Cumulative area intersected (%)
Appalachian Basin	11.97	52%	170,401	1,730,910	6%
Morondava	11.53	54%	416,921	2,147,832	7%
Red Sea Basin	11.25	56%	488,511	2,636,342	9%
Permian Basin	9.13	57%	11,646	2,647,988	9%
East Venezuela Basin	8.65	59%	122,025	2,770,013	9%
Santos Basin	8.32	60%	49,779	2,819,793	10%
West-Central Coastal	8.19	61%	114,202	2,933,994	10%
Central Sumatra Basin	8.16	63%	118,070	3,052,065	10%
Bohaiwan Basin	7.91	64%	99,992	3,152,056	11%
Burgos Basin	6.57	65%	48,485	3,200,541	11%
Greater Antilles Deformed Belt	6.33	66%	517,814	3,718,355	13%
Campos Basin	6.16	67%	33,669	3,752,024	13%
East African Rift	6.12	68%	1,326,637	5,078,661	17%
Middle Magdelena Valley Basin	5.89	69%	31,828	5,110,489	17%
Mozambique Coastal	5.54	70%	370,093	5,480,581	19%
Volga-Ural Region	5.50	71%	145,904	5,626,485	19%
Tampico-Misantla Basin	5.46	72%	27,026	5,653,511	19%
South Sumatra Basin	5.24	73%	134,523	5,788,034	20%
Parana Basin	5.20	74%	1,155,217	6,943,251	24%
Sud Province	4.93	74%	268,874	7,212,125	24%

Sedimentary Basins	Oil resources overlapped (Gbbl)	Cumulative top-priority unburnable oil resources (%)	Intersected area (km2)	Cumulative area intersected (km2)	Cumulative area intersected (%)
Tanzania Coastal	4.87	75%	243,210	7,455,336	25%
North Caspian Basin	4.70	76%	71,879	7,527,215	25%
Middle Caspian Basin	4.07	77%	178,270	7,705,485	26%
Neuquen Basin	4.05	77%	32,936	7,738,421	26%
Junggar Basin	3.74	78%	31,265	7,769,685	26%
South Caspian Basin	3.72	79%	30,950	7,800,635	26%
Kutei Basin	3.42	79%	213,249	8,013,884	27%
Qatar Arch	3.39	80%	14,082	8,027,966	27%
Putumayo-Oriente-Maranon Basin	3.35	80%	245,173	8,273,139	28%
Indus	3.31	81%	263,413	8,536,553	29%
Trias/Ghadames Basin	3.10	81%	40,645	8,577,198	29%
Essaouni Basin	2.97	82%	32,871	8,610,069	29%
Baram Delta/Brunei-Sabah Basin	2.91	82%	117,348	8,727,417	30%
Llanos Basin	2.90	83%	114,040	8,841,457	30%
Canning Basin	2.85	83%	118,211	8,959,668	30%
Malay Basin	2.74	84%	147,265	9,106,933	31%
Anglo-Paris Basin	2.74	84%	109,708	9,216,642	31%
Williston Basin	2.59	84%	65,142	9,281,783	31%
Santa Cruz-Tarija Basin	2.44	85%	187,242	9,469,025	32%
Parnaiba Basin	2.35	85%	403,677	9,872,702	33%
Bombay	2.34	86%	129,435	10,002,137	34%

Sedimentary Basins	Oil resources overlapped (Gbbl)	Cumulative top-priority unburnable oil resources (%)	Intersected area (km2)	Cumulative area intersected (km2)	Cumulative area intersected (%)
Northwest Java Basin	2.26	86%	141,795	10,143,932	34%
Denver Basin	2.21	86%	17,307	10,161,239	34%
Anglo-Dutch Basin	2.10	87%	82,135	10,243,375	35%
North Sea Graben	2.03	87%	12,938	10,256,313	35%
Gulf of Guinea	1.92	87%	47,590	10,303,903	35%
Assam	1.88	88%	90,769	10,394,673	35%
Northwest Shelf	1.86	88%	303,994	10,698,667	36%
Songliao Basin	1.82	88%	30,218	10,728,885	36%
Sirte Basin	1.74	89%	22,337	10,751,222	36%
Chad	1.68	89%	316,041	11,067,263	37%
Cauvery	1.57	89%	149,808	11,217,071	38%
Carpathian-Balkanian Basin	1.47	89%	74,299	11,291,370	38%
East Greenland Rift Basins	1.45	90%	30,261	11,321,631	38%
	...	...	...	...	...
**Total**	**608.81**	**100%**		**29,540,905.53**	**100%**

Supplementary Table 4. Global oil extraction between 2000 and 2022 disaggregated per country[16]. Cumulative production is presented in giga-barrels (thousand million barrels, Gbbl).

Country	2000	2001	2002	2003	2004	2005	2006	2007	2008	2009	2010	2011	2012	2013	2014	2015	2016	2017	2018	2019	2020	2021	2022	Total production (Gbbl)
Afghanistan	0.00	0.00	0.00	0.00	0.00	0.00	0.00	0.00	0.00	0.00	0.00	0.00	0.00	0.00	0.00	0.00	0.00	0.00	0.00	0.00	0.00	0.00	0.00	**0.00**
Albania	0.00	0.00	0.00	0.00	0.00	0.00	0.00	0.00	0.00	0.00	0.00	0.01	0.01	0.01	0.01	0.01	0.01	0.01	0.01	0.01	0.01	0.01	0.01	**0.10**
Algeria	0.52	0.55	0.59	0.65	0.67	0.71	0.72	0.72	0.71	0.70	0.69	0.68	0.68	0.64	0.63	0.66	0.62	0.60	0.58	0.57	0.51	0.52	0.54	**14.46**
American Samoa	0.00	0.00	0.00	0.00	0.00	0.00	0.00	0.00	0.00	0.00	0.00	0.00	0.00	0.00	0.00	0.00	0.00	0.00	0.00	0.00	0.00	0.00	0.00	**0.00**
Angola	0.27	0.27	0.33	0.33	0.38	0.46	0.52	0.64	0.72	0.69	0.70	0.64	0.66	0.66	0.64	0.67	0.66	0.62	0.60	0.55	0.48	0.44	0.45	**12.38**
Antarctica	0.00	0.00	0.00	0.00	0.00	0.00	0.00	0.00	0.00	0.00	0.00	0.00	0.00	0.00	0.00	0.00	0.00	0.00	0.00	0.00	0.00	0.00	0.00	**0.00**
Antigua and Barbuda	0.00	0.00	0.00	0.00	0.00	0.00	0.00	0.00	0.00	0.00	0.00	0.00	0.00	0.00	0.00	0.00	0.00	0.00	0.00	0.00	0.00	0.00	0.00	**0.00**
Argentina	0.30	0.32	0.32	0.32	0.33	0.31	0.31	0.30	0.29	0.29	0.29	0.27	0.26	0.26	0.26	0.26	0.26	0.25	0.26	0.26	0.24	0.26	0.29	**6.49**
Armenia	0.00	0.00	0.00	0.00	0.00	0.00	0.00	0.00	0.00	0.00	0.00	0.00	0.00	0.00	0.00	0.00	0.00	0.00	0.00	0.00	0.00	0.00	0.00	**0.00**
Aruba	0.00	0.00	0.00	0.00	0.00	0.00	0.00	0.00	0.00	0.00	0.00	0.00	0.00	0.00	0.00	0.00	0.00	0.00	0.00	0.00	0.00	0.00	0.00	**0.01**
Australia	0.30	0.28	0.27	0.23	0.20	0.21	0.21	0.21	0.21	0.21	0.21	0.19	0.18	0.16	0.16	0.14	0.13	0.12	0.13	0.17	0.17	0.17	0.16	**4.44**
Austria	0.01	0.01	0.01	0.01	0.01	0.01	0.01	0.01	0.01	0.01	0.01	0.01	0.01	0.01	0.01	0.01	0.01	0.01	0.01	0.01	0.01	0.01	0.01	**0.23**
Azerbaijan	0.11	0.11	0.12	0.12	0.12	0.16	0.24	0.31	0.32	0.37	0.38	0.36	0.34	0.32	0.31	0.31	0.31	0.29	0.29	0.28	0.26	0.26	0.25	**5.92**
Bahrain	0.02	0.02	0.02	0.02	0.02	0.02	0.02	0.02	0.07	0.07	0.07	0.07	0.07	0.08	0.08	0.08	0.08	0.08	0.08	0.08	0.07	0.07	0.07	**1.25**
Bangladesh	0.00	0.00	0.00	0.00	0.00	0.00	0.00	0.00	0.00	0.00	0.00	0.00	0.00	0.00	0.00	0.00	0.00	0.01	0.01	0.01	0.01	0.00	0.00	**0.06**
Barbados	0.00	0.00	0.00	0.00	0.00	0.00	0.00	0.00	0.00	0.00	0.00	0.00	0.00	0.00	0.00	0.00	0.00	0.00	0.00	0.00	0.00	0.00	0.00	**0.01**
Belarus	0.01	0.01	0.01	0.01	0.01	0.01	0.01	0.01	0.01	0.01	0.01	0.01	0.01	0.01	0.01	0.01	0.01	0.01	0.01	0.01	0.01	0.01	0.01	**0.28**
Belgium	0.00	0.00	0.00	0.00	0.00	0.00	0.00	0.00	0.00	0.00	0.01	0.01	0.01	0.01	0.01	0.01	0.01	0.01	0.01	0.01	0.01	0.01	0.01	**0.14**
Belize	0.00	0.00	0.00	0.00	0.00	0.00	0.00	0.00	0.00	0.00	0.00	0.00	0.00	0.00	0.00	0.00	0.00	0.00	0.00	0.00	0.00	0.00	0.00	**0.01**
Benin	0.00	0.00	0.00	0.00	0.00	0.00	0.00	0.00	0.00	0.00	0.00	0.00	0.00	0.00	0.00	0.00	0.00	0.00	0.00	0.00	0.00	0.00	0.00	**0.00**
Bermuda	0.00	0.00	0.00	0.00	0.00	0.00	0.00	0.00	0.00	0.00	0.00	0.00	0.00	0.00	0.00	0.00	0.00	0.00	0.00	0.00	0.00	0.00	0.00	**0.00**
Bhutan	0.00	0.00	0.00	0.00	0.00	0.00	0.00	0.00	0.00	0.00	0.00	0.00	0.00	0.00	0.00	0.00	0.00	0.00	0.00	0.00	0.00	0.00	0.00	**0.00**

Country	2000	2001	2002	2003	2004	2005	2006	2007	2008	2009	2010	2011	2012	2013	2014	2015	2016	2017	2018	2019	2020	2021	2022	Total production (Gbbl)
Bolivia	0.01	0.02	0.02	0.02	0.02	0.02	0.02	0.02	0.02	0.02	0.02	0.02	0.02	0.02	0.03	0.03	0.03	0.03	0.03	0.03	0.02	0.02	0.02	**0.50**
Bosnia and Herzegovina	0.00	0.00	0.00	0.00	0.00	0.00	0.00	0.00	0.00	0.00	0.00	0.00	0.00	0.00	0.00	0.00	0.00	0.00	0.00	0.00	0.00	0.00	0.00	**0.00**
Botswana	0.00	0.00	0.00	0.00	0.00	0.00	0.00	0.00	0.00	0.00	0.00	0.00	0.00	0.00	0.00	0.00	0.00	0.00	0.00	0.00	0.00	0.00	0.00	**0.00**
Brazil	0.56	0.57	0.64	0.67	0.67	0.74	0.79	0.83	0.89	0.93	0.99	0.98	0.97	0.98	1.08	1.16	1.18	1.22	1.25	1.34	1.38	1.35	1.39	**22.56**
British Virgin Islands	0.00	0.00	0.00	0.00	0.00	0.00	0.00	0.00	0.00	0.00	0.00	0.00	0.00	0.00	0.00	0.00	0.00	0.00	0.00	0.00	0.00	0.00	0.00	**0.00**
Brunei	0.08	0.08	0.07	0.07	0.07	0.08	0.08	0.06	0.06	0.05	0.06	0.06	0.06	0.05	0.05	0.05	0.04	0.04	0.04	0.04	0.04	0.04	0.03	**1.31**
Bulgaria	0.00	0.00	0.00	0.00	0.00	0.00	0.00	0.00	0.00	0.00	0.00	0.00	0.00	0.00	0.00	0.00	0.00	0.00	0.00	0.00	0.00	0.00	0.00	**0.03**
Burkina Faso	0.00	0.00	0.00	0.00	0.00	0.00	0.00	0.00	0.00	0.00	0.00	0.00	0.00	0.00	0.00	0.00	0.00	0.00	0.00	0.00	0.00	0.00	0.00	**0.00**
Burma	0.00	0.00	0.01	0.01	0.01	0.01	0.01	0.01	0.01	0.01	0.01	0.01	0.01	0.01	0.01	0.01	0.01	0.00	0.00	0.00	0.00	0.00	0.00	**0.14**
Burundi	0.00	0.00	0.00	0.00	0.00	0.00	0.00	0.00	0.00	0.00	0.00	0.00	0.00	0.00	0.00	0.00	0.00	0.00	0.00	0.00	0.00	0.00	0.00	**0.00**
Cabo Verde	0.00	0.00	0.00	0.00	0.00	0.00	0.00	0.00	0.00	0.00	0.00	0.00	0.00	0.00	0.00	0.00	0.00	0.00	0.00	0.00	0.00	0.00	0.00	**0.00**
Cambodia	0.00	0.00	0.00	0.00	0.00	0.00	0.00	0.00	0.00	0.00	0.00	0.00	0.00	0.00	0.00	0.00	0.00	0.00	0.00	0.00	0.00	0.00	0.00	**0.00**
Cameroon	0.03	0.03	0.03	0.02	0.02	0.03	0.03	0.03	0.03	0.03	0.02	0.02	0.02	0.02	0.03	0.04	0.03	0.03	0.03	0.03	0.02	0.02	0.02	**0.62**
Canada	1.00	1.03	1.08	1.14	1.15	1.13	1.20	1.26	1.22	1.21	1.25	1.31	1.40	1.48	1.60	1.64	1.67	1.81	1.95	2.00	1.91	2.02	2.08	**33.53**
Cayman Islands	0.00	0.00	0.00	0.00	0.00	0.00	0.00	0.00	0.00	0.00	0.00	0.00	0.00	0.00	0.00	0.00	0.00	0.00	0.00	0.00	0.00	0.00	0.00	**0.00**
Central African Republic	0.00	0.00	0.00	0.00	0.00	0.00	0.00	0.00	0.00	0.00	0.00	0.00	0.00	0.00	0.00	0.00	0.00	0.00	0.00	0.00	0.00	0.00	0.00	**0.00**
Chad	0.00	0.00	0.00	0.01	0.06	0.06	0.06	0.05	0.05	0.04	0.04	0.04	0.04	0.04	0.04	0.04	0.04	0.05	0.05	0.05	0.04	0.03	0.03	**0.88**
Chile	0.01	0.01	0.01	0.01	0.01	0.01	0.00	0.00	0.00	0.00	0.01	0.01	0.01	0.01	0.01	0.00	0.00	0.00	0.01	0.01	0.01	0.01	0.01	**0.13**
China	1.24	1.26	1.30	1.32	1.36	1.41	1.45	1.49	1.52	1.53	1.66	1.68	1.72	1.77	1.82	1.86	1.77	1.73	1.73	1.77	1.77	1.82	1.87	**36.86**
Colombia	0.26	0.23	0.21	0.20	0.20	0.20	0.20	0.20	0.22	0.25	0.29	0.34	0.36	0.38	0.37	0.38	0.33	0.32	0.33	0.33	0.30	0.28	0.28	**6.46**
Comoros	0.00	0.00	0.00	0.00	0.00	0.00	0.00	0.00	0.00	0.00	0.00	0.00	0.00	0.00	0.00	0.00	0.00	0.00	0.00	0.00	0.00	0.00	0.00	**0.00**
Congo-Brazzaville	0.11	0.10	0.09	0.09	0.09	0.09	0.09	0.08	0.09	0.10	0.13	0.11	0.10	0.09	0.09	0.09	0.07	0.09	0.13	0.12	0.11	0.10	0.10	**2.25**
Congo-Kinshasa	0.01	0.01	0.01	0.01	0.01	0.01	0.01	0.01	0.01	0.01	0.01	0.01	0.01	0.01	0.01	0.01	0.01	0.01	0.01	0.01	0.01	0.01	0.01	**0.18**

Country	2000	2001	2002	2003	2004	2005	2006	2007	2008	2009	2010	2011	2012	2013	2014	2015	2016	2017	2018	2019	2020	2021	2022	Total production (Gbbl)
Cook Islands	0.00	0.00	0.00	0.00	0.00	0.00	0.00	0.00	0.00	0.00	0.00	0.00	0.00	0.00	0.00	0.00	0.00	0.00	0.00	0.00	0.00	0.00	0.00	**0.00**
Costa Rica	0.00	0.00	0.00	0.00	0.00	0.00	0.00	0.00	0.00	0.00	0.00	0.00	0.00	0.00	0.00	0.00	0.00	0.00	0.00	0.00	0.00	0.00	0.00	**0.01**
Cote d'Ivoire	0.00	0.00	0.00	0.01	0.01	0.01	0.02	0.02	0.02	0.02	0.02	0.01	0.01	0.01	0.01	0.01	0.02	0.01	0.01	0.01	0.01	0.01	0.01	**0.31**
Croatia	0.01	0.01	0.01	0.01	0.01	0.01	0.01	0.01	0.01	0.01	0.01	0.01	0.01	0.01	0.01	0.01	0.01	0.01	0.01	0.01	0.01	0.01	0.01	**0.19**
Cuba	0.02	0.02	0.02	0.02	0.02	0.02	0.02	0.02	0.02	0.02	0.02	0.02	0.02	0.02	0.02	0.02	0.02	0.02	0.02	0.01	0.01	0.01	0.01	**0.42**
Cyprus	0.00	0.00	0.00	0.00	0.00	0.00	0.00	0.00	0.00	0.00	0.00	0.00	0.00	0.00	0.00	0.00	0.00	0.00	0.00	0.00	0.00	0.00	0.00	**0.00**
Czechia	0.00	0.00	0.00	0.01	0.01	0.01	0.01	0.01	0.00	0.00	0.01	0.01	0.01	0.00	0.01	0.01	0.01	0.00	0.00	0.00	0.00	0.00	0.00	**0.11**
Denmark	0.13	0.13	0.14	0.14	0.14	0.14	0.13	0.12	0.11	0.10	0.09	0.08	0.08	0.07	0.06	0.06	0.05	0.05	0.04	0.04	0.03	0.03	0.03	**1.96**
Djibouti	0.00	0.00	0.00	0.00	0.00	0.00	0.00	0.00	0.00	0.00	0.00	0.00	0.00	0.00	0.00	0.00	0.00	0.00	0.00	0.00	0.00	0.00	0.00	**0.00**
Dominica	0.00	0.00	0.00	0.00	0.00	0.00	0.00	0.00	0.00	0.00	0.00	0.00	0.00	0.00	0.00	0.00	0.00	0.00	0.00	0.00	0.00	0.00	0.00	**0.00**
Dominican Republic	0.00	0.00	0.00	0.00	0.00	0.00	0.00	0.00	0.00	0.00	0.00	0.00	0.00	0.00	0.00	0.00	0.00	0.00	0.00	0.00	0.00	0.00	0.00	**0.00**
Ecuador	0.14	0.15	0.14	0.15	0.19	0.20	0.20	0.19	0.19	0.18	0.18	0.18	0.19	0.19	0.20	0.20	0.20	0.20	0.19	0.19	0.18	0.17	0.18	**4.18**
Egypt	0.29	0.27	0.27	0.27	0.26	0.25	0.24	0.24	0.24	0.25	0.25	0.26	0.26	0.26	0.26	0.26	0.25	0.26	0.27	0.26	0.25	0.24	0.24	**5.90**
El Salvador	0.00	0.00	0.00	0.00	0.00	0.00	0.00	0.00	0.00	0.00	0.00	0.00	0.00	0.00	0.00	0.00	0.00	0.00	0.00	0.00	0.00	0.00	0.00	**0.01**
Equatorial Guinea	0.06	0.07	0.08	0.08	0.13	0.14	0.13	0.13	0.13	0.13	0.12	0.11	0.11	0.11	0.10	0.10	0.09	0.07	0.07	0.06	0.06	0.05	0.05	**2.17**
Eritrea	0.00	0.00	0.00	0.00	0.00	0.00	0.00	0.00	0.00	0.00	0.00	0.00	0.00	0.00	0.00	0.00	0.00	0.00	0.00	0.00	0.00	0.00	0.00	**0.00**
Estonia	0.00	0.00	0.00	0.00	0.00	0.00	0.00	0.00	0.00	0.00	0.00	0.00	0.00	0.00	0.00	0.01	0.01	0.01	0.01	0.01	0.01	0.01	0.01	**0.10**
Eswatini	0.00	0.00	0.00	0.00	0.00	0.00	0.00	0.00	0.00	0.00	0.00	0.00	0.00	0.00	0.00	0.00	0.00	0.00	0.00	0.00	0.00	0.00	0.00	**0.00**
Ethiopia	0.00	0.00	0.00	0.00	0.00	0.00	0.00	0.00	0.00	0.00	0.00	0.00	0.00	0.00	0.00	0.00	0.00	0.00	0.00	0.00	0.00	0.00	0.00	**0.00**
Falkland Islands	0.00	0.00	0.00	0.00	0.00	0.00	0.00	0.00	0.00	0.00	0.00	0.00	0.00	0.00	0.00	0.00	0.00	0.00	0.00	0.00	0.00	0.00	0.00	**0.00**
Faroe Islands	0.00	0.00	0.00	0.00	0.00	0.00	0.00	0.00	0.00	0.00	0.00	0.00	0.00	0.00	0.00	0.00	0.00	0.00	0.00	0.00	0.00	0.00	0.00	**0.00**
Fiji	0.00	0.00	0.00	0.00	0.00	0.00	0.00	0.00	0.00	0.00	0.00	0.00	0.00	0.00	0.00	0.00	0.00	0.00	0.00	0.00	0.00	0.00	0.00	**0.00**
Finland	0.00	0.00	0.00	0.00	0.00	0.00	0.00	0.00	0.00	0.00	0.01	0.00	0.01	0.01	0.01	0.01	0.00	0.00	0.01	0.01	0.01	0.01	0.01	**0.10**
France	0.03	0.03	0.03	0.03	0.03	0.03	0.03	0.03	0.03	0.03	0.05	0.04	0.04	0.04	0.04	0.04	0.04	0.04	0.04	0.04	0.04	0.04	0.04	**0.85**
French Guiana	0.00	0.00	0.00	0.00	0.00	0.00	0.00	0.00	0.00	0.00	0.00	0.00	0.00	0.00	0.00	0.00	0.00	0.00	0.00	0.00	0.00	0.00	0.00	**0.00**

Country	2000	2001	2002	2003	2004	2005	2006	2007	2008	2009	2010	2011	2012	2013	2014	2015	2016	2017	2018	2019	2020	2021	2022	Total production (Gbbl)
French Polynesia	0.00	0.00	0.00	0.00	0.00	0.00	0.00	0.00	0.00	0.00	0.00	0.00	0.00	0.00	0.00	0.00	0.00	0.00	0.00	0.00	0.00	0.00	0.00	**0.00**
Gabon	0.11	0.10	0.09	0.09	0.09	0.10	0.09	0.09	0.09	0.09	0.09	0.09	0.08	0.08	0.08	0.08	0.08	0.07	0.07	0.07	0.06	0.06	0.07	**1.92**
Gambia, The	0.00	0.00	0.00	0.00	0.00	0.00	0.00	0.00	0.00	0.00	0.00	0.00	0.00	0.00	0.00	0.00	0.00	0.00	0.00	0.00	0.00	0.00	0.00	**0.00**
Georgia	0.00	0.00	0.00	0.00	0.00	0.00	0.00	0.00	0.00	0.00	0.00	0.00	0.00	0.00	0.00	0.00	0.00	0.00	0.00	0.00	0.00	0.00	0.00	**0.01**
Germany	0.06	0.05	0.06	0.06	0.05	0.06	0.07	0.05	0.06	0.05	0.07	0.08	0.07	0.07	0.07	0.07	0.06	0.06	0.06	0.06	0.07	0.07	0.07	**1.46**
Ghana	0.00	0.00	0.00	0.00	0.00	0.00	0.00	0.00	0.00	0.00	0.00	0.03	0.03	0.04	0.04	0.04	0.04	0.06	0.06	0.07	0.07	0.07	0.07	**0.64**
Gibraltar	0.00	0.00	0.00	0.00	0.00	0.00	0.00	0.00	0.00	0.00	0.00	0.00	0.00	0.00	0.00	0.00	0.00	0.00	0.00	0.00	0.00	0.00	0.00	**0.00**
Greece	0.00	0.00	0.00	0.00	0.00	0.00	0.00	0.00	0.00	0.00	0.00	0.00	0.00	0.00	0.00	0.00	0.00	0.00	0.01	0.01	0.01	0.01	0.01	**0.08**
Greenland	0.00	0.00	0.00	0.00	0.00	0.00	0.00	0.00	0.00	0.00	0.00	0.00	0.00	0.00	0.00	0.00	0.00	0.00	0.00	0.00	0.00	0.00	0.00	**0.00**
Grenada	0.00	0.00	0.00	0.00	0.00	0.00	0.00	0.00	0.00	0.00	0.00	0.00	0.00	0.00	0.00	0.00	0.00	0.00	0.00	0.00	0.00	0.00	0.00	**0.00**
Guadeloupe	0.00	0.00	0.00	0.00	0.00	0.00	0.00	0.00	0.00	0.00	0.00	0.00	0.00	0.00	0.00	0.00	0.00	0.00	0.00	0.00	0.00	0.00	0.00	**0.00**
Guam	0.00	0.00	0.00	0.00	0.00	0.00	0.00	0.00	0.00	0.00	0.00	0.00	0.00	0.00	0.00	0.00	0.00	0.00	0.00	0.00	0.00	0.00	0.00	**0.00**
Guatemala	0.01	0.01	0.01	0.01	0.01	0.01	0.01	0.01	0.01	0.01	0.01	0.01	0.01	0.01	0.01	0.00	0.00	0.00	0.00	0.00	0.00	0.00	0.00	**0.13**
Guinea	0.00	0.00	0.00	0.00	0.00	0.00	0.00	0.00	0.00	0.00	0.00	0.00	0.00	0.00	0.00	0.00	0.00	0.00	0.00	0.00	0.00	0.00	0.00	**0.00**
Guinea-Bissau	0.00	0.00	0.00	0.00	0.00	0.00	0.00	0.00	0.00	0.00	0.00	0.00	0.00	0.00	0.00	0.00	0.00	0.00	0.00	0.00	0.00	0.00	0.00	**0.00**
Guyana	0.00	0.00	0.00	0.00	0.00	0.00	0.00	0.00	0.00	0.00	0.00	0.00	0.00	0.00	0.00	0.00	0.00	0.00	0.00	0.00	0.03	0.04	0.10	**0.17**
Haiti	0.00	0.00	0.00	0.00	0.00	0.00	0.00	0.00	0.00	0.00	0.00	0.00	0.00	0.00	0.00	0.00	0.00	0.00	0.00	0.00	0.00	0.00	0.00	**0.00**
Honduras	0.00	0.00	0.00	0.00	0.00	0.00	0.00	0.00	0.00	0.00	0.00	0.00	0.00	0.00	0.00	0.00	0.00	0.00	0.00	0.00	0.00	0.00	0.00	**0.00**
Hong Kong	0.00	0.00	0.00	0.00	0.00	0.00	0.00	0.00	0.00	0.00	0.00	0.00	0.00	0.00	0.00	0.00	0.00	0.00	0.00	0.00	0.00	0.00	0.00	**0.00**
Hungary	0.02	0.02	0.02	0.02	0.02	0.02	0.01	0.01	0.01	0.01	0.01	0.01	0.01	0.01	0.01	0.01	0.01	0.01	0.01	0.01	0.01	0.01	0.01	**0.32**
Iceland	0.00	0.00	0.00	0.00	0.00	0.00	0.00	0.00	0.00	0.00	0.00	0.00	0.00	0.00	0.00	0.00	0.00	0.00	0.00	0.00	0.00	0.00	0.00	**0.00**
India	0.28	0.29	0.30	0.30	0.31	0.30	0.32	0.32	0.32	0.32	0.35	0.36	0.36	0.36	0.36	0.36	0.36	0.36	0.36	0.35	0.32	0.33	0.32	**7.61**
Indonesia	0.56	0.52	0.49	0.45	0.43	0.40	0.38	0.36	0.37	0.37	0.37	0.36	0.35	0.34	0.33	0.31	0.34	0.33	0.33	0.33	0.33	0.31	0.30	**8.68**
Iran	1.37	1.39	1.29	1.40	1.50	1.55	1.51	1.47	1.52	1.52	1.56	1.56	1.31	1.22	1.27	1.30	1.62	1.74	1.66	1.20	1.09	1.26	1.34	**32.67**
Iraq	0.94	0.88	0.74	0.48	0.74	0.69	0.73	0.77	0.87	0.88	0.89	0.97	1.10	1.12	1.24	1.49	1.64	1.65	1.71	1.75	1.52	1.51	1.66	**25.98**
Ireland	0.00	0.00	0.00	0.00	0.00	0.00	0.00	0.00	0.00	0.00	0.00	0.00	0.00	0.00	0.00	0.00	0.00	0.00	0.00	0.00	0.00	0.00	0.00	**0.00**

Country	2000	2001	2002	2003	2004	2005	2006	2007	2008	2009	2010	2011	2012	2013	2014	2015	2016	2017	2018	2019	2020	2021	2022	Total production (Gbbl)
Israel	0.00	0.00	0.00	0.00	0.00	0.00	0.00	0.00	0.00	0.00	0.00	0.00	0.00	0.00	0.00	0.00	0.00	0.00	0.00	0.00	0.00	0.00	0.00	**0.05**
Italy	0.05	0.04	0.05	0.05	0.06	0.06	0.06	0.06	0.06	0.05	0.06	0.06	0.06	0.07	0.06	0.07	0.05	0.05	0.05	0.05	0.05	0.05	0.05	**1.26**
Jamaica	0.00	0.00	0.00	0.00	0.00	0.00	0.00	0.00	0.00	0.00	0.00	0.00	0.00	0.00	0.00	0.00	0.00	0.00	0.00	0.00	0.00	0.00	0.00	**0.02**
Japan	0.04	0.04	0.04	0.04	0.05	0.05	0.05	0.05	0.05	0.04	0.05	0.04	0.04	0.04	0.04	0.04	0.03	0.04	0.04	0.04	0.04	0.04	0.04	**0.95**
Jordan	0.00	0.00	0.00	0.00	0.00	0.00	0.00	0.00	0.00	0.00	0.00	0.00	0.00	0.00	0.00	0.00	0.00	0.00	0.00	0.00	0.00	0.00	0.00	**0.00**
Kazakhstan	0.26	0.31	0.35	0.39	0.45	0.49	0.51	0.53	0.52	0.56	0.59	0.60	0.59	0.61	0.63	0.64	0.62	0.69	0.72	0.72	0.68	0.68	0.67	**12.78**
Kenya	0.00	0.00	0.00	0.00	0.00	0.00	0.00	0.00	0.00	0.00	0.00	0.00	0.00	0.00	0.00	0.00	0.00	0.00	0.00	0.00	0.00	0.00	0.00	**0.00**
Kiribati	0.00	0.00	0.00	0.00	0.00	0.00	0.00	0.00	0.00	0.00	0.00	0.00	0.00	0.00	0.00	0.00	0.00	0.00	0.00	0.00	0.00	0.00	0.00	**0.00**
Kosovo	0.00	0.00	0.00	0.00	0.00	0.00	0.00	0.00	0.00	0.00	0.00	0.00	0.00	0.00	0.00	0.00	0.00	0.00	0.00	0.00	0.00	0.00	0.00	**0.00**
Kuwait	0.80	0.78	0.74	0.83	0.92	0.98	0.98	0.95	1.00	0.91	0.89	0.99	1.03	1.06	1.06	1.12	1.16	1.10	1.12	1.10	1.00	0.99	1.10	**22.60**
Kyrgyzstan	0.00	0.00	0.00	0.00	0.00	0.00	0.00	0.00	0.00	0.00	0.00	0.00	0.00	0.00	0.00	0.00	0.00	0.00	0.00	0.00	0.00	0.00	0.00	**0.01**
Laos	0.00	0.00	0.00	0.00	0.00	0.00	0.00	0.00	0.00	0.00	0.00	0.00	0.00	0.00	0.00	0.00	0.00	0.00	0.00	0.00	0.00	0.00	0.00	**0.00**
Latvia	0.00	0.00	0.00	0.00	0.00	0.00	0.00	0.00	0.00	0.00	0.00	0.00	0.00	0.00	0.00	0.00	0.00	0.00	0.00	0.00	0.00	0.00	0.00	**0.01**
Lebanon	0.00	0.00	0.00	0.00	0.00	0.00	0.00	0.00	0.00	0.00	0.00	0.00	0.00	0.00	0.00	0.00	0.00	0.00	0.00	0.00	0.00	0.00	0.00	**0.00**
Lesotho	0.00	0.00	0.00	0.00	0.00	0.00	0.00	0.00	0.00	0.00	0.00	0.00	0.00	0.00	0.00	0.00	0.00	0.00	0.00	0.00	0.00	0.00	0.00	**0.00**
Liberia	0.00	0.00	0.00	0.00	0.00	0.00	0.00	0.00	0.00	0.00	0.00	0.00	0.00	0.00	0.00	0.00	0.00	0.00	0.00	0.00	0.00	0.00	0.00	**0.00**
Libya	0.54	0.52	0.50	0.54	0.58	0.63	0.68	0.70	0.71	0.67	0.67	0.19	0.56	0.38	0.21	0.18	0.17	0.33	0.38	0.44	0.15	0.46	0.39	**10.61**
Lithuania	0.00	0.00	0.00	0.00	0.00	0.00	0.00	0.00	0.00	0.00	0.00	0.00	0.00	0.00	0.00	0.00	0.00	0.00	0.00	0.00	0.00	0.00	0.00	**0.08**
Luxembourg	0.00	0.00	0.00	0.00	0.00	0.00	0.00	0.00	0.00	0.00	0.00	0.00	0.00	0.00	0.00	0.00	0.00	0.00	0.00	0.00	0.00	0.00	0.00	**0.00**
Macau	0.00	0.00	0.00	0.00	0.00	0.00	0.00	0.00	0.00	0.00	0.00	0.00	0.00	0.00	0.00	0.00	0.00	0.00	0.00	0.00	0.00	0.00	0.00	**0.00**
Madagascar	0.00	0.00	0.00	0.00	0.00	0.00	0.00	0.00	0.00	0.00	0.00	0.00	0.00	0.00	0.00	0.00	0.00	0.00	0.00	0.00	0.00	0.00	0.00	**0.00**
Malawi	0.00	0.00	0.00	0.00	0.00	0.00	0.00	0.00	0.00	0.00	0.00	0.00	0.00	0.00	0.00	0.00	0.00	0.00	0.00	0.00	0.00	0.00	0.00	**0.00**
Malaysia	0.28	0.27	0.29	0.30	0.29	0.29	0.27	0.28	0.28	0.26	0.27	0.24	0.25	0.24	0.24	0.26	0.28	0.28	0.27	0.26	0.24	0.22	0.22	**6.08**
Maldives	0.00	0.00	0.00	0.00	0.00	0.00	0.00	0.00	0.00	0.00	0.00	0.00	0.00	0.00	0.00	0.00	0.00	0.00	0.00	0.00	0.00	0.00	0.00	**0.00**
Mali	0.00	0.00	0.00	0.00	0.00	0.00	0.00	0.00	0.00	0.00	0.00	0.00	0.00	0.00	0.00	0.00	0.00	0.00	0.00	0.00	0.00	0.00	0.00	**0.00**
Malta	0.00	0.00	0.00	0.00	0.00	0.00	0.00	0.00	0.00	0.00	0.00	0.00	0.00	0.00	0.00	0.00	0.00	0.00	0.00	0.00	0.00	0.00	0.00	**0.00**
Martinique	0.00	0.00	0.00	0.00	0.00	0.00	0.00	0.00	0.00	0.00	0.00	0.00	0.00	0.00	0.00	0.00	0.00	0.00	0.00	0.00	0.00	0.00	0.00	**0.00**

Country	2000	2001	2002	2003	2004	2005	2006	2007	2008	2009	2010	2011	2012	2013	2014	2015	2016	2017	2018	2019	2020	2021	2022	Total production (Gbbl)
Mauritania	0.00	0.00	0.00	0.00	0.00	0.00	0.01	0.01	0.00	0.00	0.00	0.00	0.00	0.00	0.00	0.00	0.00	0.00	0.00	0.00	0.00	0.00	0.00	**0.04**
Mauritius	0.00	0.00	0.00	0.00	0.00	0.00	0.00	0.00	0.00	0.00	0.00	0.00	0.00	0.00	0.00	0.00	0.00	0.00	0.00	0.00	0.00	0.00	0.00	**0.00**
Mexico	1.26	1.30	1.31	1.39	1.40	1.38	1.35	1.28	1.16	1.09	1.09	1.08	1.07	1.06	1.03	0.95	0.90	0.82	0.76	0.70	0.71	0.72	0.74	**24.57**
Micronesia	0.00	0.00	0.00	0.00	0.00	0.00	0.00	0.00	0.00	0.00	0.00	0.00	0.00	0.00	0.00	0.00	0.00	0.00	0.00	0.00	0.00	0.00	0.00	**0.00**
Moldova	0.00	0.00	0.00	0.00	0.00	0.00	0.00	0.00	0.00	0.00	0.00	0.00	0.00	0.00	0.00	0.00	0.00	0.00	0.00	0.00	0.00	0.00	0.00	**0.00**
Mongolia	0.00	0.00	0.00	0.00	0.00	0.00	0.00	0.00	0.00	0.00	0.00	0.00	0.00	0.01	0.01	0.01	0.01	0.01	0.01	0.01	0.01	0.01	0.01	**0.08**
Montserrat	0.00	0.00	0.00	0.00	0.00	0.00	0.00	0.00	0.00	0.00	0.00	0.00	0.00	0.00	0.00	0.00	0.00	0.00	0.00	0.00	0.00	0.00	0.00	**0.00**
Morocco	0.00	0.00	0.00	0.00	0.00	0.00	0.00	0.00	0.00	0.00	0.00	0.00	0.00	0.00	0.00	0.00	0.00	0.00	0.00	0.00	0.00	0.00	0.00	**0.00**
Mozambique	0.00	0.00	0.00	0.00	0.00	0.00	0.00	0.00	0.00	0.00	0.00	0.00	0.00	0.00	0.00	0.00	0.00	0.00	0.00	0.00	0.00	0.00	0.00	**0.00**
Namibia	0.00	0.00	0.00	0.00	0.00	0.00	0.00	0.00	0.00	0.00	0.00	0.00	0.00	0.00	0.00	0.00	0.00	0.00	0.00	0.00	0.00	0.00	0.00	**0.00**
Nauru	0.00	0.00	0.00	0.00	0.00	0.00	0.00	0.00	0.00	0.00	0.00	0.00	0.00	0.00	0.00	0.00	0.00	0.00	0.00	0.00	0.00	0.00	0.00	**0.00**
Nepal	0.00	0.00	0.00	0.00	0.00	0.00	0.00	0.00	0.00	0.00	0.00	0.00	0.00	0.00	0.00	0.00	0.00	0.00	0.00	0.00	0.00	0.00	0.00	**0.00**
Netherlands	0.03	0.03	0.04	0.04	0.04	0.03	0.03	0.03	0.03	0.02	0.02	0.02	0.03	0.03	0.03	0.03	0.02	0.02	0.02	0.03	0.03	0.03	0.03	**0.67**
Netherlands Antilles	0.00	0.00	0.00	0.00	0.00	0.00	0.00	0.00	0.00	0.00	0.00	0.00	0.00	0.00	0.00	0.00	0.00	0.00	0.00	0.00	0.00	0.00	0.00	**0.00**
New Caledonia	0.00	0.00	0.00	0.00	0.00	0.00	0.00	0.00	0.00	0.00	0.00	0.00	0.00	0.00	0.00	0.00	0.00	0.00	0.00	0.00	0.00	0.00	0.00	**0.00**
New Zealand	0.02	0.02	0.01	0.01	0.01	0.01	0.01	0.02	0.02	0.02	0.02	0.02	0.02	0.02	0.02	0.02	0.02	0.01	0.01	0.01	0.01	0.01	0.01	**0.34**
Nicaragua	0.00	0.00	0.00	0.00	0.00	0.00	0.00	0.00	0.00	0.00	0.00	0.00	0.00	0.00	0.00	0.00	0.00	0.00	0.00	0.00	0.00	0.00	0.00	**0.00**
Niger	0.00	0.00	0.00	0.00	0.00	0.00	0.00	0.00	0.00	0.00	0.00	0.00	0.01	0.01	0.01	0.01	0.00	0.00	0.00	0.00	0.00	0.00	0.00	**0.06**
Nigeria	0.79	0.83	0.78	0.83	0.85	0.96	0.89	0.86	0.79	0.81	0.90	0.92	0.92	0.86	0.88	0.82	0.71	0.74	0.73	0.74	0.68	0.60	0.52	**18.39**
Niue	0.00	0.00	0.00	0.00	0.00	0.00	0.00	0.00	0.00	0.00	0.00	0.00	0.00	0.00	0.00	0.00	0.00	0.00	0.00	0.00	0.00	0.00	0.00	**0.00**
North Korea	0.00	0.00	0.00	0.00	0.00	0.00	0.00	0.00	0.00	0.00	0.00	0.00	0.00	0.00	0.00	0.00	0.00	0.00	0.00	0.00	0.00	0.00	0.00	**0.00**
North Macedonia	0.00	0.00	0.00	0.00	0.00	0.00	0.00	0.00	0.00	0.00	0.00	0.00	0.00	0.00	0.00	0.00	0.00	0.00	0.00	0.00	0.00	0.00	0.00	**0.00**
Northern Mariana Islands	0.00	0.00	0.00	0.00	0.00	0.00	0.00	0.00	0.00	0.00	0.00	0.00	0.00	0.00	0.00	0.00	0.00	0.00	0.00	0.00	0.00	0.00	0.00	**0.00**
Norway	1.22	1.25	1.22	1.19	1.17	1.09	1.02	0.94	0.90	0.86	0.78	0.75	0.70	0.67	0.69	0.71	0.73	0.72	0.68	0.64	0.73	0.74	0.70	**20.09**

Country	2000	2001	2002	2003	2004	2005	2006	2007	2008	2009	2010	2011	2012	2013	2014	2015	2016	2017	2018	2019	2020	2021	2022	Total production (Gbbl)
Oman	0.35	0.33	0.33	0.30	0.28	0.28	0.27	0.26	0.28	0.30	0.32	0.33	0.34	0.34	0.35	0.36	0.37	0.36	0.36	0.36	0.35	0.36	0.39	**7.56**
Pakistan	0.02	0.02	0.02	0.02	0.02	0.02	0.03	0.03	0.02	0.02	0.03	0.03	0.03	0.03	0.04	0.03	0.03	0.04	0.04	0.04	0.03	0.04	0.04	**0.68**
Palestinian Territories	0.00	0.00	0.00	0.00	0.00	0.00	0.00	0.00	0.00	0.00	0.00	0.00	0.00	0.00	0.00	0.00	0.00	0.00	0.00	0.00	0.00	0.00	0.00	**0.00**
Panama	0.00	0.00	0.00	0.00	0.00	0.00	0.00	0.00	0.00	0.00	0.00	0.00	0.00	0.00	0.00	0.00	0.00	0.00	0.00	0.00	0.00	0.00	0.00	**0.00**
Papua New Guinea	0.03	0.02	0.02	0.02	0.02	0.01	0.02	0.01	0.02	0.01	0.01	0.01	0.01	0.01	0.02	0.02	0.02	0.02	0.01	0.01	0.01	0.01	0.01	**0.37**
Paraguay	0.00	0.00	0.00	0.00	0.00	0.00	0.00	0.00	0.00	0.00	0.00	0.00	0.00	0.00	0.00	0.00	0.00	0.00	0.00	0.00	0.00	0.00	0.00	**0.02**
Peru	0.04	0.04	0.04	0.03	0.03	0.04	0.04	0.04	0.04	0.05	0.06	0.06	0.06	0.06	0.07	0.06	0.05	0.05	0.05	0.05	0.05	0.05	0.04	**1.10**
Philippines	0.00	0.00	0.00	0.01	0.01	0.01	0.01	0.01	0.01	0.01	0.01	0.01	0.01	0.01	0.02	0.01	0.01	0.01	0.01	0.01	0.01	0.01	0.01	**0.20**
Poland	0.01	0.01	0.01	0.01	0.01	0.01	0.01	0.01	0.01	0.01	0.01	0.01	0.02	0.02	0.02	0.02	0.02	0.01	0.02	0.01	0.01	0.01	0.01	**0.31**
Portugal	0.00	0.00	0.00	0.00	0.00	0.00	0.00	0.00	0.00	0.00	0.00	0.00	0.00	0.00	0.00	0.00	0.00	0.00	0.00	0.00	0.00	0.00	0.00	**0.07**
Puerto Rico	0.00	0.00	0.00	0.00	0.00	0.00	0.00	0.00	0.00	0.00	0.00	0.00	0.00	0.00	0.00	0.00	0.00	0.00	0.00	0.00	0.00	0.00	0.00	**0.00**
Qatar	0.32	0.32	0.32	0.37	0.42	0.46	0.47	0.49	0.54	0.54	0.61	0.66	0.73	0.73	0.70	0.68	0.67	0.65	0.66	0.64	0.64	0.67	0.68	**13.00**
Reunion	0.00	0.00	0.00	0.00	0.00	0.00	0.00	0.00	0.00	0.00	0.00	0.00	0.00	0.00	0.00	0.00	0.00	0.00	0.00	0.00	0.00	0.00	0.00	**0.00**
Romania	0.05	0.05	0.05	0.05	0.05	0.05	0.04	0.04	0.04	0.04	0.04	0.04	0.04	0.04	0.04	0.04	0.03	0.03	0.03	0.03	0.03	0.03	0.03	**0.91**
Russia	2.45	2.61	2.80	3.12	3.38	3.47	3.55	3.63	3.60	3.67	3.76	3.80	3.87	3.93	3.96	4.03	4.10	4.10	4.16	4.19	3.83	3.93	3.99	**83.93**
Rwanda	0.00	0.00	0.00	0.00	0.00	0.00	0.00	0.00	0.00	0.00	0.00	0.00	0.00	0.00	0.00	0.00	0.00	0.00	0.00	0.00	0.00	0.00	0.00	**0.00**
Saint Helena	0.00	0.00	0.00	0.00	0.00	0.00	0.00	0.00	0.00	0.00	0.00	0.00	0.00	0.00	0.00	0.00	0.00	0.00	0.00	0.00	0.00	0.00	0.00	**0.00**
Saint Kitts and Nevis	0.00	0.00	0.00	0.00	0.00	0.00	0.00	0.00	0.00	0.00	0.00	0.00	0.00	0.00	0.00	0.00	0.00	0.00	0.00	0.00	0.00	0.00	0.00	**0.00**
Saint Lucia	0.00	0.00	0.00	0.00	0.00	0.00	0.00	0.00	0.00	0.00	0.00	0.00	0.00	0.00	0.00	0.00	0.00	0.00	0.00	0.00	0.00	0.00	0.00	**0.00**
Saint Pierre and Miquelon	0.00	0.00	0.00	0.00	0.00	0.00	0.00	0.00	0.00	0.00	0.00	0.00	0.00	0.00	0.00	0.00	0.00	0.00	0.00	0.00	0.00	0.00	0.00	**0.00**
Saint Vincent/Grenadines	0.00	0.00	0.00	0.00	0.00	0.00	0.00	0.00	0.00	0.00	0.00	0.00	0.00	0.00	0.00	0.00	0.00	0.00	0.00	0.00	0.00	0.00	0.00	**0.00**
Samoa	0.00	0.00	0.00	0.00	0.00	0.00	0.00	0.00	0.00	0.00	0.00	0.00	0.00	0.00	0.00	0.00	0.00	0.00	0.00	0.00	0.00	0.00	0.00	**0.00**
Sao Tome and Principe	0.00	0.00	0.00	0.00	0.00	0.00	0.00	0.00	0.00	0.00	0.00	0.00	0.00	0.00	0.00	0.00	0.00	0.00	0.00	0.00	0.00	0.00	0.00	**0.00**

Country	2000	2001	2002	2003	2004	2005	2006	2007	2008	2009	2010	2011	2012	2013	2014	2015	2016	2017	2018	2019	2020	2021	2022	Total production (Gbbl)
Saudi Arabia	3.46	3.34	3.22	3.68	3.94	4.20	4.05	3.92	4.17	3.76	3.56	4.02	4.19	4.11	4.14	4.29	4.44	4.32	4.42	4.19	3.96	3.95	4.43	**91.77**
Senegal	0.00	0.00	0.00	0.00	0.00	0.00	0.00	0.00	0.00	0.00	0.00	0.00	0.00	0.00	0.00	0.00	0.00	0.00	0.00	0.00	0.00	0.00	0.00	**0.00**
Serbia	0.00	0.00	0.00	0.00	0.00	0.00	0.01	0.01	0.01	0.01	0.01	0.01	0.01	0.01	0.01	0.01	0.01	0.01	0.01	0.01	0.01	0.01	0.01	**0.12**
Seychelles	0.00	0.00	0.00	0.00	0.00	0.00	0.00	0.00	0.00	0.00	0.00	0.00	0.00	0.00	0.00	0.00	0.00	0.00	0.00	0.00	0.00	0.00	0.00	**0.00**
Sierra Leone	0.00	0.00	0.00	0.00	0.00	0.00	0.00	0.00	0.00	0.00	0.00	0.00	0.00	0.00	0.00	0.00	0.00	0.00	0.00	0.00	0.00	0.00	0.00	**0.00**
Singapore	0.00	0.01	0.00	0.00	0.00	0.00	0.00	0.00	0.01	0.01	0.01	0.01	0.01	0.01	0.01	0.01	0.01	0.01	0.01	0.01	0.01	0.01	0.01	**0.17**
Slovakia	0.00	0.00	0.00	0.00	0.00	0.00	0.00	0.00	0.00	0.00	0.00	0.00	0.00	0.00	0.00	0.00	0.00	0.00	0.00	0.00	0.00	0.00	0.00	**0.08**
Slovenia	0.00	0.00	0.00	0.00	0.00	0.00	0.00	0.00	0.00	0.00	0.00	0.00	0.00	0.00	0.00	0.00	0.00	0.00	0.00	0.00	0.00	0.00	0.00	**0.00**
Solomon Islands	0.00	0.00	0.00	0.00	0.00	0.00	0.00	0.00	0.00	0.00	0.00	0.00	0.00	0.00	0.00	0.00	0.00	0.00	0.00	0.00	0.00	0.00	0.00	**0.00**
Somalia	0.00	0.00	0.00	0.00	0.00	0.00	0.00	0.00	0.00	0.00	0.00	0.00	0.00	0.00	0.00	0.00	0.00	0.00	0.00	0.00	0.00	0.00	0.00	**0.00**
South Africa	0.07	0.08	0.08	0.07	0.09	0.08	0.07	0.07	0.07	0.07	0.07	0.07	0.06	0.06	0.06	0.04	0.04	0.04	0.04	0.04	0.04	0.04	0.04	**1.39**
South Korea	0.01	0.01	0.01	0.01	0.01	0.01	0.01	0.01	0.01	0.02	0.02	0.02	0.03	0.03	0.03	0.03	0.03	0.03	0.03	0.04	0.04	0.04	0.04	**0.50**
South Sudan	0.00	0.00	0.00	0.00	0.00	0.00	0.00	0.00	0.00	0.00	0.00	0.00	0.01	0.04	0.05	0.06	0.06	0.05	0.06	0.06	0.06	0.06	0.06	**0.56**
Spain	0.01	0.01	0.01	0.01	0.01	0.01	0.01	0.01	0.01	0.01	0.02	0.02	0.02	0.02	0.02	0.02	0.02	0.03	0.03	0.03	0.03	0.03	0.03	**0.42**
Sri Lanka	0.00	0.00	0.00	0.00	0.00	0.00	0.00	0.00	0.00	0.00	0.00	0.00	0.00	0.00	0.00	0.00	0.00	0.00	0.00	0.00	0.00	0.00	0.00	**0.00**
Sudan	0.07	0.08	0.09	0.10	0.13	0.13	0.14	0.17	0.18	0.18	0.18	0.17	0.03	0.05	0.04	0.04	0.04	0.04	0.03	0.03	0.02	0.02	0.02	**1.96**
Suriname	0.00	0.00	0.00	0.00	0.00	0.00	0.00	0.00	0.01	0.01	0.01	0.01	0.01	0.01	0.01	0.01	0.01	0.01	0.01	0.01	0.01	0.01	0.00	**0.11**
Sweden	0.00	0.00	0.00	0.00	0.00	0.00	0.00	0.00	0.00	0.00	0.00	0.00	0.00	0.00	0.00	0.00	0.00	0.00	0.01	0.00	0.00	0.00	0.00	**0.08**
Switzerland	0.00	0.00	0.00	0.00	0.00	0.00	0.00	0.00	0.00	0.00	0.00	0.00	0.00	0.00	0.00	0.00	0.00	0.00	0.00	0.00	0.00	0.00	0.00	**0.02**
Syria	0.20	0.19	0.19	0.19	0.18	0.17	0.16	0.15	0.15	0.15	0.15	0.14	0.06	0.03	0.01	0.01	0.01	0.01	0.01	0.01	0.02	0.03	0.03	**2.24**
Taiwan	0.00	0.00	0.00	0.00	0.00	0.01	0.01	0.01	0.01	0.01	0.01	0.01	0.01	0.01	0.01	0.01	0.01	0.01	0.01	0.01	0.01	0.01	0.01	**0.16**
Tajikistan	0.00	0.00	0.00	0.00	0.00	0.00	0.00	0.00	0.00	0.00	0.00	0.00	0.00	0.00	0.00	0.00	0.00	0.00	0.00	0.00	0.00	0.00	0.00	**0.00**
Tanzania	0.00	0.00	0.00	0.00	0.00	0.00	0.00	0.00	0.00	0.00	0.00	0.00	0.00	0.00	0.00	0.00	0.00	0.00	0.00	0.00	0.00	0.00	0.00	**0.00**
Thailand	0.07	0.07	0.08	0.10	0.09	0.11	0.13	0.13	0.14	0.15	0.15	0.16	0.18	0.18	0.18	0.19	0.19	0.19	0.19	0.19	0.18	0.17	0.15	**3.37**
The Bahamas	0.00	0.00	0.00	0.00	0.00	0.00	0.00	0.00	0.00	0.00	0.00	0.00	0.00	0.00	0.00	0.00	0.00	0.00	0.00	0.00	0.00	0.00	0.00	**0.00**
Timor-Leste	0.00	0.00	0.00	0.00	0.02	0.03	0.04	0.03	0.04	0.04	0.03	0.04	0.04	0.03	0.02	0.02	0.01	0.01	0.01	0.01	0.01	0.01	0.01	**0.41**

Country	2000	2001	2002	2003	2004	2005	2006	2007	2008	2009	2010	2011	2012	2013	2014	2015	2016	2017	2018	2019	2020	2021	2022	Total production (Gbbl)
Togo	0.00	0.00	0.00	0.00	0.00	0.00	0.00	0.00	0.00	0.00	0.00	0.00	0.00	0.00	0.00	0.00	0.00	0.00	0.00	0.00	0.00	0.00	0.00	**0.00**
Tonga	0.00	0.00	0.00	0.00	0.00	0.00	0.00	0.00	0.00	0.00	0.00	0.00	0.00	0.00	0.00	0.00	0.00	0.00	0.00	0.00	0.00	0.00	0.00	**0.00**
Trinidad and Tobago	0.05	0.05	0.05	0.06	0.06	0.06	0.07	0.06	0.06	0.06	0.05	0.04	0.04	0.04	0.04	0.04	0.04	0.04	0.03	0.03	0.03	0.03	0.03	**1.03**
Tunisia	0.03	0.03	0.03	0.03	0.03	0.03	0.03	0.03	0.03	0.03	0.03	0.02	0.02	0.02	0.02	0.01	0.02	0.02	0.01	0.01	0.01	0.02	0.01	**0.53**
Turkiye	0.02	0.02	0.02	0.02	0.02	0.02	0.02	0.02	0.02	0.02	0.02	0.02	0.02	0.02	0.02	0.02	0.02	0.02	0.03	0.03	0.03	0.03	0.03	**0.49**
Turkmenistan	0.06	0.06	0.07	0.07	0.08	0.07	0.06	0.07	0.07	0.07	0.08	0.08	0.09	0.09	0.10	0.10	0.10	0.10	0.10	0.09	0.09	0.09	0.09	**1.88**
Turks and Caicos Islands	0.00	0.00	0.00	0.00	0.00	0.00	0.00	0.00	0.00	0.00	0.00	0.00	0.00	0.00	0.00	0.00	0.00	0.00	0.00	0.00	0.00	0.00	0.00	**0.00**
Tuvalu	0.00	0.00	0.00	0.00	0.00	0.00	0.00	0.00	0.00	0.00	0.00	0.00	0.00	0.00	0.00	0.00	0.00	0.00	0.00	0.00	0.00	0.00	0.00	**0.00**
U.S. Pacific Islands	0.00	0.00	0.00	0.00	0.00	0.00	0.00	0.00	0.00	0.00	0.00	0.00	0.00	0.00	0.00	0.00	0.00	0.00	0.00	0.00	0.00	0.00	0.00	**0.00**
U.S. Territories	0.00	0.00	0.00	0.00	0.00	0.00	0.00	0.00	0.00	0.00	0.00	0.00	0.00	0.00	0.00	0.00	0.00	0.00	0.00	0.00	0.00	0.00	0.00	**0.00**
U.S. Virgin Islands	0.01	0.01	0.01	0.01	0.01	0.01	0.01	0.01	0.01	0.01	0.01	0.01	0.00	0.00	0.00	0.00	0.00	0.00	0.00	0.00	0.00	0.00	0.00	**0.07**
Uganda	0.00	0.00	0.00	0.00	0.00	0.00	0.00	0.00	0.00	0.00	0.00	0.00	0.00	0.00	0.00	0.00	0.00	0.00	0.00	0.00	0.00	0.00	0.00	**0.00**
Ukraine	0.03	0.03	0.03	0.03	0.04	0.04	0.04	0.04	0.04	0.03	0.03	0.03	0.03	0.03	0.02	0.02	0.02	0.02	0.02	0.02	0.02	0.02	0.01	**0.64**
United Arab Emirates	0.94	0.91	0.87	0.97	1.01	1.04	1.08	1.08	1.11	1.02	1.03	1.17	1.24	1.26	1.29	1.34	1.38	1.36	1.38	1.51	1.38	1.38	1.55	**27.29**
United Kingdom	0.94	0.95	0.94	0.87	0.76	0.68	0.62	0.62	0.58	0.55	0.51	0.42	0.36	0.34	0.33	0.37	0.39	0.38	0.42	0.43	0.40	0.34	0.32	**12.51**
United States	3.31	3.27	3.28	3.20	3.18	3.04	3.03	3.09	3.13	3.33	3.54	3.71	4.07	4.52	5.17	5.53	5.42	5.72	6.54	7.11	6.79	6.93	7.38	**104.29**
Uruguay	0.00	0.00	0.00	0.00	0.00	0.00	0.00	0.00	0.00	0.00	0.00	0.00	0.00	0.00	0.00	0.00	0.00	0.00	0.00	0.00	0.00	0.00	0.00	**0.01**
Uzbekistan	0.06	0.06	0.06	0.06	0.05	0.05	0.04	0.04	0.04	0.03	0.02	0.02	0.02	0.03	0.02	0.02	0.02	0.02	0.02	0.02	0.01	0.01	0.02	**0.73**
Vanuatu	0.00	0.00	0.00	0.00	0.00	0.00	0.00	0.00	0.00	0.00	0.00	0.00	0.00	0.00	0.00	0.00	0.00	0.00	0.00	0.00	0.00	0.00	0.00	**0.00**
Venezuela	1.26	1.22	1.07	0.94	1.04	1.05	1.00	0.98	0.99	0.99	0.95	0.98	0.98	0.98	0.98	0.94	0.85	0.75	0.56	0.34	0.20	0.22	0.26	**19.54**
Vietnam	0.12	0.13	0.12	0.13	0.15	0.14	0.13	0.12	0.11	0.12	0.11	0.12	0.13	0.13	0.12	0.13	0.12	0.10	0.09	0.09	0.08	0.08	0.07	**2.64**
Wake Island	0.00	0.00	0.00	0.00	0.00	0.00	0.00	0.00	0.00	0.00	0.00	0.00	0.00	0.00	0.00	0.00	0.00	0.00	0.00	0.00	0.00	0.00	0.00	**0.00**
Western Sahara	0.00	0.00	0.00	0.00	0.00	0.00	0.00	0.00	0.00	0.00	0.00	0.00	0.00	0.00	0.00	0.00	0.00	0.00	0.00	0.00	0.00	0.00	0.00	**0.00**

Country	2000	2001	2002	2003	2004	2005	2006	2007	2008	2009	2010	2011	2012	2013	2014	2015	2016	2017	2018	2019	2020	2021	2022	Total production (Gbbl)
Yemen	0.16	0.16	0.16	0.16	0.15	0.15	0.14	0.12	0.11	0.10	0.10	0.08	0.06	0.05	0.05	0.02	0.01	0.01	0.02	0.02	0.02	0.02	0.02	**1.87**
Zambia	0.00	0.00	0.00	0.00	0.00	0.00	0.00	0.00	0.00	0.00	0.00	0.00	0.00	0.00	0.00	0.00	0.00	0.00	0.00	0.00	0.00	0.00	0.00	**0.00**
Zimbabwe	0.00	0.00	0.00	0.00	0.00	0.00	0.00	0.00	0.00	0.00	0.00	0.00	0.00	0.00	0.00	0.00	0.00	0.00	0.00	0.00	0.00	0.00	0.00	**0.00**
**World**	**28.37**	**28.35**	**28.14**	**29.06**	**30.48**	**31.08**	**31.13**	**31.15**	**31.67**	**31.29**	**31.87**	**32.30**	**33.13**	**33.33**	**34.27**	**35.27**	**35.44**	**35.70**	**36.68**	**36.59**	**34.26**	**34.94**	**36.51**	**751.00**

References

1. O'Callaghan-Gordo, C., Orta-Martínez, M. & Kogevinas, M. Health effects of non-occupational exposure to oil extraction. *Environ Health* **15**, 56 (2016).

2. Konkel, L. Salting the Earth: The Environmental Impact of Oil and Gas Wastewater Spills. *Environ Health Perspect* **124**, (2016).

3. Johnston, J. E., Lim, E. & Roh, H. Impact of upstream oil extraction and environmental public health: A review of the evidence. *Science of the Total Environment* vol. 657 (2019).

4. Deziel, N. C. *et al.* Unconventional oil and gas development and health outcomes: A scoping review of the epidemiological research. *Environmental Research* vol. 182 (2020).

5. Jernelv, A. The threats from oil spills: Now, then, and in the future. *Ambio* **39**, 353–366 (2010).

6. Fry, M. Urban gas drilling and distance ordinances in the Texas Barnett Shale. *Energy Policy* **62**, (2013).

7. McKenzie, L. M., Witter, R. Z., Newman, L. S. & Adgate, J. L. Human health risk assessment of air emissions from development of unconventional natural gas resources. *Science of the Total Environment* **424**, (2012).

8. Coons, T. & Walker, R. *Community Health Risk Analysis of oil and gas industry impacts in Garfield County*. https://www.garfield-county.com/air-quality/ (2008).

9. Osborn, S. G., Vengosh, A., Warner, N. R. & Jackson, R. B. Methane contamination of drinking water accompanying gas-well drilling and hydraulic fracturing. *Proc Natl Acad Sci U S A* **108**, (2011).

10. Hill, E. L. Shale gas development and infant health: Evidence from Pennsylvania. *J Health Econ* **61**, (2018).

11. Meng, Q. Spatial analysis of environment and population at risk of natural gas fracking in the state of Pennsylvania, USA. *Science of the Total Environment* **515–516**, (2015).

12. O'Callaghan-Gordo, C. *et al.* Blood lead levels in indigenous peoples living close to oil extraction areas in the Peruvian Amazon. *Environ Int* **154**, 106639 (2021).

13. González Alonso, S., Esteban-Hernández, J., Valcárcel Rivera, Y., Hernández-Barrera, V. & Gil De Miguel, A. Water pollution in sources close to oil-producing fields of Bolivia. *Revista Panamericana de Salud Publica/Pan American Journal of Public Health* (2010).

14. Yusta-García, R., Orta-Martínez, M., Mayor, P., González-Crespo, C. & Rosell-Melé, A. Water contamination from oil extraction activities in Northern Peruvian Amazonian rivers. *Environmental Pollution* **225**, 370–380 (2017).

15. Welsby, D., Price, J., Pye, S. & Ekins, P. Unextractable fossil fuels in a 1.5 °C world. *Nature* **597**, (2021).

16. United States Energy Information Administration (EIA). International. Petroleum and other liquids. Production. https://www.eia.gov/international/data/world/petroleum-and-other-liquids/annual-refined-petroleum-products-consumption?pd=5&p=0000000000000000000000000000000000vg&u=0&f=A&v=mapbubble&a=-

&i=none&vo=value&t=C&g=00000000000000000000000000000000000000000000000001&l=249-ruvvvvvfvtvnvv1urvvvvfvvvvvvfvvvou20evvvvvvvvvnvvvvs&s=94694400000&e=1640995200000&vb=170&ev=false (2022).
