# Supplementary material for: The atlas of unburnable oil for supply-side climate policies
Source: Nat Commun. 2024 Mar 14;15:2318. doi: 10.1038/s41467-024-46340-6 (PMC10940309; doi:10.1038/s41467-024-46340-6)
Supplement: Supplementary file 3 — Reporting Summary [file 41467_2024_46340_MOESM3_ESM.pdf]

nature portfolio

Corresponding author(s): Martí Orta-Martínez

Last updated by author(s): Dec 21, 2023

Reporting Summary

Nature Portfolio wishes to improve the reproducibility of the work that we publish. This form provides structure for consistency and transparency in reporting. For further information on Nature Portfolio policies, see our Editorial Policies and the Editorial Policy Checklist.

Statistics

For all statistical analyses, confirm that the following items are present in the figure legend, table legend, main text, or Methods section.

n/a	Confirmed	
☒	☐	The exact sample size (*n*) for each experimental group/condition, given as a discrete number and unit of measurement
☒	☐	A statement on whether measurements were taken from distinct samples or whether the same sample was measured repeatedly
☒	☐	The statistical test(s) used AND whether they are one- or two-sided<br>*Only common tests should be described solely by name; describe more complex techniques in the Methods section.*
☒	☐	A description of all covariates tested
☒	☐	A description of any assumptions or corrections, such as tests of normality and adjustment for multiple comparisons
☒	☐	A full description of the statistical parameters including central tendency (e.g. means) or other basic estimates (e.g. regression coefficient) AND variation (e.g. standard deviation) or associated estimates of uncertainty (e.g. confidence intervals)
☒	☐	For null hypothesis testing, the test statistic (e.g. *F*, *t*, *r*) with confidence intervals, effect sizes, degrees of freedom and *P* value noted<br>*Give P values as exact values whenever suitable.*
☒	☐	For Bayesian analysis, information on the choice of priors and Markov chain Monte Carlo settings
☒	☐	For hierarchical and complex designs, identification of the appropriate level for tests and full reporting of outcomes
☒	☐	Estimates of effect sizes (e.g. Cohen's *d*, Pearson's *r*), indicating how they were calculated

Our web collection on statistics for biologists contains articles on many of the points above.

Software and code

Policy information about availability of computer code

Data collection	Our work is based on the spatial analysis of secondary data. We compiled global datasets from governmental agencies, multilateral institutions, non-governmental organizations and standard-setting organizations. Details on data collection and management are provided in the 'Methods' section of the article. Commercial, open source or custom codes were not used to collect data in this study
Data analysis	The spatial analyses were conducted using open source and commercial Geographical Information Systems (GIS): QGIS Desktop 3.22.5 Białowieża with GRASS 8.2.1, ArcGIS Desktop 10.6 and ArcGIS Pro 3.0.3. We have not used any custom code or mathematical algorithm, but the conventional tools provided by the GIS software used.

For manuscripts utilizing custom algorithms or software that are central to the research but not yet described in published literature, software must be made available to editors and reviewers. We strongly encourage code deposition in a community repository (e.g. GitHub). See the Nature Portfolio guidelines for submitting code & software for further information.

Data

Policy information about availability of data

All manuscripts must include a data availability statement. This statement should provide the following information, where applicable:

- Accession codes, unique identifiers, or web links for publicly available datasets
- A description of any restrictions on data availability
- For clinical datasets or third party data, please ensure that the statement adheres to our policy

We follow the FAIR principles (i.e. Findable, Accessible, Interoperable and Reusable data) and the principles of EU Open Science Policy. All the data generated in this study have been deposited in the Digital Repository of the corresponding author's university (University of Barcelona): https://doi.org/10.34810/data891. Source data are provided with this paper. The sources and hyperlinks to the raw data used in this study are provided in Source Data file.

The following open-access databases/datasets have been used in the study:

Data on conventional oil resources outside the United States:
USGS World Petroleum Assessment (WPA) 2000- United States Geological Service (USGS). World Petroleum Assessment 2000. U.S. Geological Survey Digital Data Series DDS60. Data Series. https://pubs.usgs.gov/dds/dds-060/
Undiscovered Conventional Oil and Gas Resources of the World 2012- United States Geological Service (USGS). Supporting data for the U.S. Geological Survey 2012 world assessment of undiscovered oil and gas resources. https://pubs.usgs.gov/publication/ds69FF doi:https://doi.org/10.3133/ds69FF
World Undiscovered Asessments (2012-2022) https://www.usgs.gov/centers/central-energy-resources-science-center/science/world-oil-and-gas-resource-assessments#publications

Data on conventional oil resources in the United States:
USGS NOGA Resources Update- United States Geological Service (USGS). USGS National Assessment of oil and gas resources update (March, 2013). https://certmapper.cr.usgs.gov/data/noga00/natl/tabular/2013/Summary_13_Final.xls
USGS national assessment reports on undiscovered oil and gas resources (2013-2022): United States Geological Service (USGS). National Oil and Gas Assessment. https://www.usgs.gov/programs/energy-resources-program/science/science-topics/national-oil-and-gas-assessment.

Geospatial data on conventional oil basins outside the United States:
United States Geological Service. World Petroleum Assessment 2000. Oil basins outside the United States: http://certmapper.cr.usgs.gov/data/wep/dds60/wep_prvg.zip
United States Geological Service. Undiscovered Conventional Oil and Gas Resources of the World 2012. Oil basins outside the United States. Supporting data for the U.S. Geological Survey 2012 world assessment of undiscovered oil and gas resources. https://pubs.usgs.gov/publication/ds69FF (2013) doi:https://doi.org/10.3133/ds69FF.

Geospatial data on conventional oil basins in the United States:
National USGS: United States Geological Service (USGS). 1995 National Assessment of United States Oil and Gas Resources. Circular 1118. https://pubs.usgs.gov/circ/1995/1118/report.pdf (1995).
NOGA Province Boundaries update from 2012- United States Geological Service (USGS). National Oil and Gas Assessment Province Boundaries through 2012. https://certmapper.cr.usgs.gov/data/noga00/natl/spatial/geodatabase/usprov12gdb.zip (2012).

Biodiversity hotspots:
Critical Ecosystem Partnership Fund (CEPF). Biodiversity Hotspots 2016. https://www.arcgis.com/home/item.html?id=fb8ec2af7cfc40c7af89d9b7e922d4d8 (2018).

Centres of diversity for endemic species:
Jenkins, C. N., Pimm, S. L. & Joppa, L. N. Conservation Science Around the World Website. Global patterns of terrestrial vertebrate diversity and conservation GIS database. http://www4.ncsu.edu/~cnjenki2/Projects.html. (2013).
Jenkins, C. N. & Van Houtan, K. S. DRYAD. Data from: Global and regional priorities for marine biodiversity protection [Dataset]. https://datadryad.org/stash/dataset/doi:10.5061/dryad.3mn1t (2017) doi:https://doi.org/10.5061/dryad.3mn1t.

World Protected Areas:
UNEP-WCMC. Protected Planet. World Protected Areas Database. https://www.protectedplanet.net/en/thematic-areas/wdpa?tab=WDPA

Urban Areas:
Center for Sustainability and the Global Environment (SAGE). Nelson Institute for Environmental Studies. UW-Madison. Global Maps of Urban Extent from Satellite Data. https://sage.nelson.wisc.edu/data-and-models/datasets/#urbanextent

Rural population density:
FAO. Rural population density 2000. Food Insecurity, Poverty and Environment Global GIS Database. http://www.fao.org/geonetwork/srv/en/resources.get?id=14052&fname=Map_2_2.zip&access=private (2007).

Presence of Indigenous Peoples in Voluntary Isolation:
RAISG. RAISG. Cartographic data. Visualization of geospatial information about the Amazon. Download. https://www.amazoniasocioambiental.org/en/maps/ (2022).
Survival International. Survival International. Indigenous peoples living in voluntary isolation in the South-East Asia. http://www.uncontactedtribes.org/where.

Research involving human participants, their data, or biological material

Policy information about studies with human participants or human data. See also policy information about sex, gender (identity/presentation), and sexual orientation and race, ethnicity and racism.

Reporting on sex and gender	Information not collected
Reporting on race, ethnicity, or other socially relevant groupings	Information not collected
Population characteristics	Information not collected
Recruitment	Information not collected
Ethics oversight	Our institutions have no mandatory Ethics review for studies using only secondary and anonymous data

Note that full information on the approval of the study protocol must also be provided in the manuscript.

Field-specific reporting

Please select the one below that is the best fit for your research. If you are not sure, read the appropriate sections before making your selection.

☐ Life sciences ☐ Behavioural & social sciences ☒ Ecological, evolutionary & environmental sciences

For a reference copy of the document with all sections, see nature.com/documents/nr-reporting-summary-flat.pdf

Ecological, evolutionary & environmental sciences study design

All studies must disclose on these points even when the disclosure is negative.

Study description

We develop an integrated spatial assessment model that uses estimates and locations of the world's conventional oil resources and socio-environmental criteria to construct the first global atlas of unburnable oil resources

Research sample

GIS datasets on oil reserves and resources

The two most common metrics to report the availability of oil are ‘oil reserves’ and ‘oil resources’. Oil reserves are the quantity of remaining oil that is recoverable under current economic conditions. Oil resources (or, remaining ultimately recoverable oil resources) denote the quantity of remaining oil that is recoverable over time with both current and future technology, irrespective of current economic conditions73. Thus, reserves are a subset of resources. Furthermore, oil is usually also categorized as ‘conventional’ and ‘unconventional’. Here, following Ref.8, we define oil with density lower than water (often standardized as ‘10° API’) as conventional (i.e. oil, LTO, and NGL) and the rest, unconventional (i.e. natural bitumen, extra-heavy oil, and kerogen oil). However, it is important to note that there is no full consensus among on whether LTO should be considered conventional or unconventional oil and some of the world energy standard setting institutions (i.e. USGS, SPE, EIA and IEA) define LTO as unconventional59–62. According to these institutions, unconventional oil lacks the porosity and permeability of conventional reservoirs required to flow without stimulation and require specialized extraction technology (e.g. hydraulic fracturing stimulation for LTO).

Although reserves are more likely to be extracted than resources, our analysis is focused on resources for several reasons. From a policy perspective, an analysis of resources is a first logical step towards the fulfilment of the Paris Agreement commitments since an analysis of resources can guide investment in oil exploration. At the same time, reserve volume estimates fluctuate over time depending on prices, the cost of available technologies, the development of new oil extraction technologies, new discoveries, and strategic overestimation by rights holders74,75. There is much uncertainty about future energy prices which heavily depend on some key political choices, including the climate mitigation policies adopted by governments around the world. Ultimately, reserves depend crucially on climate policies, turning an analysis focusing on reserves into a questionable policy tool. Nevertheless, we acknowledge that an analysis of reserves is an important next step in the research on unextractable fossil fuels and our methodology could be adapted to construct atlases for unburnable fossil fuel reserves.Using a remaining carbon budget of 580 GtCO2, ref.8 established that 744 Gbbl (58%) of current oil reserves, both conventional and unconventional, should remain unburned, while 1,823 Gbbl (71%) and 1,513 Gbbl (99%) of conventional and unconventional oil resources, respectively, were considered unburnable. This distribution of unburnable oil categories was based on the production costs of different resources, taking into account extraction, refining, and transport costs. Thus, according to ref.8, the overwhelming majority of unconventional oil should remain unburned because its production was considerably less economic than the production of other oil categories. Building on ref.8, our study focuses on the identification of those conventional oil resources that should remain unburned according to social and biological criteria

For that purpose, we compiled a dataset of global conventional oil resources based on data from the United States Geological Survey (USGS). The dataset was put together following the method described by ref.41 For world conventional oil resources, we used data from the World Oil and Gas Assessments produced by the USGS World Energy Project, in particular the USGS World Petroleum Assessment (WPA) 2000 (USGS, 2000) and 2012 (USGS, 2012a) and, 27 regional USGS assessment reports on undiscovered oil and gas resources in priority geologic provinces in the World published between 2012 and September 2022 (https://www.usgs.gov/centers/central-energy-resources-science-center/science/world-oil-and-gas-resource-assessments#publications). The USGS WPA 2000 provides estimates of the quantities of remaining technically recoverable conventional oil resources outside the United States that had the potential to be added to reserves from 1995 to 2025 (i.e. discovered reserves + contingent resources, according to the definition of SPE62). We used spatial and tabular data on remaining oil and remaining NGL (i.e. quantities of conventional oil and NGL

excluding reported cumulative volume of oil and NGL that had been already produced) from the WPA 2000 ("wep_prvg.shp" geospatial data set). From the USGS WPA 2012, we have retrieved estimates of the quantities of undiscovered (or 'prospective' according to the definition of SPE62) conventional oil resources). Specifically, we used spatial and tabular data on undiscovered technically recoverable oil resources (i.e., the oil that could be produced using available technology and industry practices regardless of economic or accessibility considerations) from the WPA 2012 ("Province Summary.xls").

When individual regional reports for specific sedimentary basins have been issued after 2012, undiscovered conventional oil volumes from the WPA 2012 have been updated. Thus, conventional oil resources volumes have been calculated by adding remaining discovered conventional oil resources as in the WPA 2000, undiscovered conventional oil resources as in the WPA 2012, and individual USGS assessments for sedimentary basins from 2012 onwards. For USA conventional oil resources, we used data from National Oil and Gas Assessments (NOGA) produced by the USGS. We used tabular data on conventional oil resources volumes for USA geological provinces from the USGS NOGA Resources Update (March, 2013) (USGS, 2013) and 6 USGS national assessment reports on undiscovered oil and gas resources published between 2013 and September 2022. The spatial data for USA geological provinces were acquired from the USGS NOGA 1995 (USGS, 1995) and the NOGA Province Boundaries update from 2012 (USGS, 2012b). These are the most accurate and up to date open-access and available georeferenced datasets on conventional oil resources at the global level. However, it is important to note that these estimates of the global conventional oil resources are subject to some degree of uncertainty (as demonstrated by the variation of the estimates over time and across different sources)76. One limitation of our data is that we do not account for (a) conventional oil extraction between 2000 and 2022 (2013-2022 for USA) and, (b) discoveries made between 2000 and 2012+ (i.e. resources added to contingent /discovered resources from prospective/ undiscovered resources –according to the definition of SPE- between the WPA 2000 and the WPA 2012 or the individual USGS assessments published for each particular sedimentary basin from 2012 onwards). Although there are no available data on the conventional oil extracted in each basin between 2000 and 2022, 751 Gbbl of conventional oil have been extracted globally in this period77 -see Supplementary Table 3 in the Supplementary Information. While this omission tends to overestimate the amount existing resources, the additions to the contingent resources category from the prospective resources category between 2000 and 2012+ produces an underestimation. It is worth mentioning that between 2000 and 2022, global reserves have increased by 685 Gbbl -585 Gbbl between 2000 and 2012-78. Future research, when updated data on oil extraction and discovered resources become available at the basin level, could gauge the implications of updated oil resource estimates for the spatial distribution of unburnable oil.

There are significant differences between the georeferenced resources we use, and the non-georeferenced tabular data provided by other sources and datasets. However, we believe the two are close enough in both definition and size to be a meaningful proxy. We put together georeferenced data for 2,276 Gbbl of conventional oil resources, while ref.8 provided an estimate of 2,575 Gbbl. These differences are explained by the dearth of accessible and up-to-date spatial data. Ref.8 estimated that 1,823 Gbbl (71%) of the conventional oil resources should be considered unburnable and that, therefore, 752 Gbbl (29%) could be burned. Out of the 2,276 Gbbl of georeferenced conventional oil resources, 1,524 Gbbl (2,276 minus 752 Gbbl) should be left untapped and we use socio-environmental variables to rank and prioritize these resources. If any of the other conventional oil resources not considered in this study (because of the unavailability of georeferenced data) are burned, a larger portion of the conventional oil resources for which we have georeferenced data should be left untapped in order to limit global warming to 1.5°C. The global energy systems model used by ref.8 assumed a 2018–2100 carbon budget of 580 GtCO2.

To identify the unburnable oil resources whose conservation would generate substantial collateral socio-environmental benefits, our spatial analysis builds upon the cost-optimal distribution of unburnable fuels among coal, gas and oil proposed by ref.8 (i.e., a key assumption of our spatial analysis is that, as set by ref.8, 71% of the conventional oil resources should remain unextracted by 2050, as well as 81% of conventional gas resources, 99% of unconventional oil resources, 93% of unconventional gas resources and 97% of coal).

The equivalences used to convert different types of fossil fuels into CO2 emission are the following: 0.43 metric tons CO2/barrel of oil equivalent; 0.0548 metric tons CO2/Mcf (thousand cubic feet) of natural gas; 9.05 x 10-4 metric tons CO2/pound of coal79,80.

Regarding our spatial unit of analysis, we have used the 313Assessment Units of the WPA 2012 (USGS, 2012), located in one of the 1,71 geologic provinces of the world, and 67 USA geological provinces (USGS, 1995, 2012b). Geological provinces are USGS-defined areas having characteristic dimensions of hundreds to thousands of square kilometers encompassing a natural geologic entity (i.e. sedimentary basin, thrust belt, delta), or some combination of contiguous geologic entities. We are aware that the use of finer spatial units of analysis, such as oil fields or reservoirs, would be a significant methodological improvement, because it would bring mapping to scales comparable with regional decisions on which oil fields and reservoirs should be kept untapped. However, these data are inaccessible for scientific purposes, or are available at a substantial price. Access to complementary spatial datasets on gas and coal resources, but also on other up-to-date spatial data on conventional oil categories, is needed to provide a complete overview for policy makers.

Georeferenced datasets for biological criteria: Global biodiversity, conservation priorities and Natural Protected Areas

Different datasets have been used to identify oil resources that overlap global biodiversity conservation priorities66. We have used two different schemes: biodiversity hotspots63 and centres of diversity for endemic terrestrial and marine species64,65. To align our proposal for unburnable conventional oil resources with current efforts and measures for biodiversity protection worldwide, we have used a spatial database of the natural protected areas81. Furthermore, to rank additional oil resources beyond the top-priority unburnable ones, we have also used global data on the geographic distribution of terrestrial and marine endemic species64,65.

Biodiversity Hotspots is the best-known and the most widely accepted Global Biodiversity Conservation Priorities scheme. Biodiversity Hotspots were defined by Norman Myers63,82,83 and reassessed up to the current 36 biodiversity hotspots designated in 2016. To qualify as a hotspot, a region has to contain at least 1,500 species of vascular plants as endemics and have 30 percent or less of its original vegetation remaining. Among conservation biologists there is broad consensus on the use of the Biodiversity Hotspot approach and it has been adopted by standard-setting organization worldwide such as Conservation International, the Critical Ecosystem Partnership Fund (CEPF), and the Global Environment Facility (GEF). We retrieved the georeferenced data from CEPF (Version 2016.1. 25 April 2016).

Although existing global conservation priorities are based on biodiversity hotspots, there are also other approaches. Thus, making use of species-distribution databases, such as the Global Amphibian Assessment or the Global Mammal Assessment84,85, Jenkins et al reassessed terrestrial64 and marine65 conservation priority areas. Using new data on >21,000 species of mammals, amphibians, and birds, and focusing on endemic species, Jenkins et al. identified conservation priority areas for vertebrates64. Similary, ref.65 assessed global marine biodiversity conservation priority areas by evaluating the geographic ranges of 4,352 marine species of 9 different taxa: plants, fish, echinoderms, crustaceans, cnidarians, mollusks, mammals, reptiles, and birds65. Jenkins's terrestrial and marine priority areas do not only reflect today's improved knowledge of biodiversity, but also consider both marine and terrestrial ecosystems. Instead, plant-based Biodiversity Hotspots are circumscribed to terrestrial areas. Therefore, we also used these centres

of diversity for endemic terrestrial and marine species as an alternative scheme of global biodiversity conservation priorities to identify the unburnable conventional oil reserves and resources.

GIS data on biodiversity conservation priority areas for terrestrial vertebrates and on the distribution of >21,000 species of mammals, amphibians, and birds were retrieved from the Conservation Science Around the World Website (accessed February 2019). GIS data on geographic ranges of marine species were retrieved from the Dryad Digital Repository.

The dataset we used for the planet's protected area system was the World Database on Protected Areas (WDPA)81. The WDPA is a joint project between the United Nations Environment Programme (UNEP) and the International Union for Conservation of Nature (IUCN). It is compiled and managed by the UNEP World Conservation Monitoring Centre (UNEP-WCMC). The WDPA is the most up to date and comprehensive global database of marine and terrestrial protected areas, updated monthly in collaboration with governments, non-governmental organisations, academia and industry. It is made available online through Protected Planet. The WDPA version we used was updated in July 2020. The WDPA includes all six IUCN Protected Area Management Categories, from strictly protected IUCN categories (I–IV) to the lowest protection level (V-VI), and protected areas for which there is no IUCN Protected Area Management Category.

GIS datasets for social criteria: Indigenous peoples in voluntary isolation, urban areas and rural population densities

To assess the social value of conventional oil resources we used the following datasets: urban areas, territories of Indigenous Peoples in Voluntary Isolation, and rural population density.

Regarding data on urban areas, we used the MODIS 500-m global map of urban extent produced by the University of Wisconsin-Madison, Boston University and the MODIS Land Group86,87. 'Urban areas' are identified through remote sensing based on physical attributes (not on population densities). The 'MODIS 500-m global map of urban extent' defines 'urban areas' as pixels that are dominated by the built environment. 'Built environment' includes all non-vegetative, human-constructed land covers, such as buildings, roads, runways, etc., and 'dominated' implies coverage greater than or equal to 50% of the pixel. All these areas, including a 10 km buffer around them, were designated as irreconcilable with oil extraction. Although these 'urban areas' also include industrial areas (i.e areas that could arguably be suitable for oil extraction), they are, to our knowledge, the best proxy for global data on densely populated areas (both rural and urban) The 10km buffer was set considering health risks associated with oil extraction (see 'Supplementary Information' for a detailed explanation of the 10km safe distance).

For the areas beyond the 'built environment', we used a dataset for rural population density: the Rural population density map of the Food Insecurity, Poverty and Environment Global GIS Database (FGGD)69. FGGD is a global database maintained by the Food and Agricultural Organization (FAO) to analyse food insecurity and poverty in relation to the environment. The FGGD rural population density map provides estimates of the global population distribution in 2015. It is a global raster datalayer with a resolution of 5 arc-minutes. Each pixel classified as non-built environment by the urban area boundaries map contains the number of persons per square kilometer, aggregated from the 30 arc-second datalayer. The method used by FAO to generate this datalayer is described in ref.88 .

We also used the presence of Indigenous Peoples in voluntary isolation as a criterion for primary selection of unburnable conventional oil resources. Indigenous Peoples in voluntary isolation do not maintain sustained contact with non-indigenous populations, and generally avoid it because most of the previous contacts have been violent and have had serious consequences for them89. Given their situation of isolation with respect to the non-indigenous societies, they do not have the immunological defenses to relatively common diseases, and contagions often have devastating effects on them causing outbreaks with high rates of mortality. Groups in voluntary isolation or initial contact exist in the Amazon as well as Paraguay (Chaco), India (Andaman islands), and Papua New Guinea. The dataset used for territories of Indigenous peoples in Voluntary Isolation was pieced together from two different institutions: the Amazon Georeferenced Socio-Environmental Information Network (RAISG, in its Spanish acronym) and Survival International. RAISG is a consortium of civil society organizations from the Amazon countries that produces comprehensive socio-environmental geospatial data on Amazonia. GIS data from RAISG, downloaded from here, was used for the territories of Indigenous peoples living in voluntary isolation in the Amazon. GIS data from Survival International was used for the territories of Indigenous peoples living in voluntary isolation in the South-East Asia. Survival International, founded in 1969, is one of the key civil society organizations defending the rights of Indigenous Peoples. Data was downloaded from here.

Sampling strategy	Spatial analysis including all the available global data. Without sampling strategy
Data collection	The process of data acquisition involved the collection of open-access tabular and geospatial data retrieved from numerous assessment reports and on-line sources (listed in the data availability statement and in the 'methods' section of the manuscript). Data collection of conventional oil resources, as well as biological and social datasets, was undertaken by Martí Orta-Martínez, Gorka Muñoa and Guillem Rius-Taberner. Tabular data compilation of conventional oil resources entailed the manual input of volumes of technically recoverable conventional oil for each sedimentary basin into an attribute table and the merging with the geospatial data files.
Timing and spatial scale	The secondary datasets used in this study were initially retrieved from online open-access sources in 2017. These datasets were updated subsequently until August 2023 whenever new versions were available. Specific dates for each dataset are detailed in the 'methods' section of the manuscript.
Data exclusions	No
Reproducibility	No experimental findings
Randomization	No allocation to groups
Blinding	This study is based on the spatial analysis of secondary datasets of global conventional oil resources and environmental and social criteria. Blinding was not relevant to the study since no experimental design was used to analyse the data.

Did the study involve field work? ☐ Yes ☒ No

Reporting for specific materials, systems and methods

We require information from authors about some types of materials, experimental systems and methods used in many studies. Here, indicate whether each material, system or method listed is relevant to your study. If you are not sure if a list item applies to your research, read the appropriate section before selecting a response.

Materials & experimental systems

n/a	Involved in the study
☒	☐ Antibodies
☒	☐ Eukaryotic cell lines
☒	☐ Palaeontology and archaeology
☒	☐ Animals and other organisms
☒	☐ Clinical data
☒	☐ Dual use research of concern
☒	☐ Plants

Methods

n/a	Involved in the study
☒	☐ ChIP-seq
☒	☐ Flow cytometry
☒	☐ MRI-based neuroimaging

Plants

Seed stocks	No plants used in the study
Novel plant genotypes	No plants used in the study
Authentication	No plants used in the study
